# The Type III Secretion Effector CteG Mediates Host Cell Lytic Exit of *Chlamydia trachomatis*

**DOI:** 10.3389/fcimb.2022.902210

**Published:** 2022-07-08

**Authors:** Inês Serrano Pereira, Sara Vilela Pais, Vítor Borges, Maria José Borrego, João Paulo Gomes, Luís Jaime Mota

**Affiliations:** ^1^ Associate Laboratory i4HB - Institute for Health and Bioeconomy, NOVA School of Science and Technology, NOVA University Lisbon, Caparica, Portugal; ^2^ UCIBIO – Applied Molecular Biosciences Unit, Department of Life Sciences, NOVA School of Science and Technology, NOVA University Lisbon, Caparica, Portugal; ^3^ Bioinformatics Unit, Department of Infectious Diseases, National Institute of Health Dr. Ricardo Jorge (INSA), Lisbon, Portugal

**Keywords:** host-pathogen interactions, pathogen egress, *Chlamydia trachomatis*, type III secretion, effectors

## Abstract

*Chlamydia trachomatis* is an obligate intracellular bacterium causing ocular and urogenital infections in humans that are a significant burden worldwide. The completion of its characteristic infectious cycle relies on the manipulation of several host cell processes by numerous chlamydial type III secretion effector proteins. We previously identified the *C. trachomatis* CteG effector and showed it localizes at the host cell plasma membrane at late stages of infection. Here, we showed that, from 48 h post-infection, mammalian cells infected by wild-type *C. trachomatis* contained more infectious chlamydiae in the culture supernatant than cells infected by a CteG-deficient strain. This phenotype was CteG-dependent as it could be complemented in cells infected by the CteG-deficient strain carrying a plasmid encoding CteG. Furthermore, we detected a CteG-dependent defect on host cell cytotoxicity, indicating that CteG mediates chlamydial lytic exit. Previous studies showed that Pgp4, a global regulator of transcription encoded in the *C. trachomatis* virulence plasmid, also mediates chlamydial lytic exit. However, by using *C. trachomatis* strains encoding or lacking Pgp4, we showed that production and localization of CteG are not regulated by Pgp4. A *C. trachomatis* strain lacking both CteG and Pgp4 was as defective in promoting host cell cytotoxicity as mutant strains lacking only CteG or Pgp4. Furthermore, CteG overproduction in a plasmid suppressed the host cell cytotoxic defect of CteG- and Pgp4-deficient chlamydiae. Overall, we revealed the first chlamydial type III secretion effector involved in host cell lytic exit. Our data indicates that CteG and Pgp4 participate in a single cascade of events, but involving multiple layers of regulation, leading to lysis of host cells and release of the infectious chlamydiae.

## Introduction

The ability of intracellular pathogens to establish a successful infection is largely dependent on their capacity to proliferate and spread within their hosts. Because such pathogens spend part of their life cycle in an intracellular niche, exit from host cells is a crucial event for dissemination. Pathogens can exit host cells by inducing programmed cell death, such as necroptosis or pyroptosis (Lindgren et al., 1996; Uwamahoro et al., 2014; Dallenga et al., 2017), or by destruction of host cell membranes mediated directly by proteins from the pathogens and unrelated with different modes of programmed host cell death (Kafsack et al., 2009; Glushakova et al., 2018). However, they can also egress from host cells while leaving them intact (Chen et al., 2004; Johnston and May, 2010; Kim et al., 2013; Weddle and Agaisse, 2018). Despite the numerous studies reporting host cell exit routes of intracellular pathogens, many of the mechanisms driving these phenomena are still unclear or poorly described.

The obligate intracellular bacterial pathogen *Chlamydia trachomatis* has been shown to egress from host cells by lytic and non-lytic mechanisms (Hybiske and Stephens, 2007). This pathogen infects exclusively humans, causing trachoma (Taylor et al., 2014) and sexually transmitted diseases, including lymprogranuloma venereum (LGV) (Batteiger, 2012; Elwell et al., 2016). *C. trachomatis* is characterized by a developmental/infectious cycle involving the inter-conversion between an infectious but non-replicative form (elementary body), and a non-infectious but replicative form (reticulate body), which culminates with the release of infectious elementary bodies. During most of the cycle, the chlamydiae remain within a membrane-bound compartment (known as inclusion) and use a type III secretion (T3S) system to deliver virulence proteins, known as effectors, into host cells (Mueller et al., 2014; Elwell et al., 2016; Bugalhão and Mota, 2019). *C. trachomatis* T3S effectors comprise inclusion membrane proteins (Incs), which insert in the inclusion membrane and have been shown to interfere with several host cell processes, such as vesicular and non-vesicular trafficking, cytoskeleton dynamics and immune response, or to promote inclusion stability, acquisition of host lipids, and bacterial host cell exit (Mirrashidi et al., 2015; Elwell et al., 2016; Bugalhão and Mota, 2019). Other non-Inc T3S effectors were shown to be delivered to the outside of the inclusion and act, for example, on chlamydial invasion or at the modulation of host cell vesicular trafficking (Bugalhão and Mota, 2019). *C. trachomatis* proteins that are not T3S substrates have also been detected in the host cell cytosol, such as the chlamydial protease/proteasome-like activity factor (CPAF) (Zhong, 2011).

In the extrusion pathway leading to *C. trachomatis* egress, the entire and intact inclusion is released from the host cell into the extracellular space in a process where actin polymerization, small GTPases of the Rho family, and myosin II have been shown to play a role (Hybiske and Stephens, 2007). *C. trachomatis* Incs CT228 and MrcA regulate extrusion by controlling the activity of myosin II (Lutter et al., 2013; Nguyen et al., 2018; Shaw et al., 2018). On the other hand, *C. trachomatis* lytic exit involves sequential rupture of chlamydial inclusion and host cell plasma membranes with concomitant release of elementary bodies and host cell death (Hybiske and Stephens, 2007). This pathway was shown to depend on intracellular calcium levels and proteases, specifically cysteine proteases (Hybiske and Stephens, 2007). *C. trachomatis* lytic exit depends on a virulence plasmid that is present in most *Chlamydia* species (Yang et al., 2015). Although the *Chlamydia* plasmid is dispensable for chlamydial growth in cell culture infection models, it contributes for pathogenicity in mice infection models (Zhong, 2017). One of the eight plasmid-encoded open reading frames (Pgp4), regulates the expression of several chromosomal and plasmid genes (Carlson et al., 2008; Song et al., 2013; Patton et al., 2018), and a Pgp4-deficient *C. trachomatis* strain fails to exit host cells as a plasmid-free *C. trachomatis* strain (Yang et al., 2015). In the presence of a compound described as an inhibitor of the T3S system, wild-type *C. trachomatis* also fails to exit host cells (Yang et al., 2015). This led to the hypothesis that Pgp4-dependent lytic exit might involve a chromosomal T3S effector gene. Furthermore, a *cpaf* null mutant *C. trachomatis* is also defective in lysis of infected cultured cells (Yang et al., 2015). However, expression of CPAF is not regulated by Pgp4 (Patton et al., 2018), and it has been suggested that CPAF could play an indirect role in *C. trachomatis* lytic exit (Yang et al., 2015).

In previous studies, we identified CT105/CTL0360 as a *C. trachomatis* T3S protein delivered into the host cell cytoplasm during infection (da Cunha et al., 2014; Pais et al., 2019). The protein was named CteG for *C. trachomatis* effector associated with the Golgi, as it localizes in the host cell Golgi from 16-20 h post-infection (Pais et al., 2019). As host cell infection progresses, CteG starts localizing at the host cell plasma membrane and from 30-40 h post-infection this is its predominant localization (Pais et al., 2019). It is unknown how this change in the localization of CteG occurs, but it is independent of intact host cell microfilaments or microtubules (Pais et al., 2019). Furthermore, although *C. trachomatis* infection promotes the redistribution of the Golgi complex around the inclusion (Heuer et al., 2009), this is not dependent on CteG (Pais et al., 2019). Infection of HeLa cells with a *C. trachomatis cteG* mutant that we generated resulted in smaller inclusions than those displayed by the parental strain, but this defect could not be complemented by *cteG* in *trans* (Pais et al., 2019). Here, we pursued the characterization of the *C. trachomatis cteG* mutant strain and show that a function of CteG is to mediate *C. trachomatis* lytic exit from host cells.

## Materials and Methods

### Cell Culture

HeLa 229 and Vero cells (from the European Collection of Authenticated Cell Culture; ECACC) were passaged in 4.5 g/L glucose, L-glutamine Dulbecco’s Modified Eagle’s Medium (DMEM; Corning) supplemented with heat-inactivated 10% (v/v) fetal bovine serum (FBS; Thermo Fisher Scientific) at 37°C in a humidified atmosphere of 5% (v/v) CO_2_. Cell cultures were regularly tested for *Mycoplasma* by conventional PCR, as described (Uphoff and Drexler, 2011).

### DNA Manipulation, Primers, and Plasmids

The plasmids used in this work, and a description of their construction and main characteristics are specified in **Supplementary Table S1**. The DNA oligonucleotides used in plasmid construction and in other molecular biology procedures are listed in **Supplementary Table S2**. Plasmids were constructed using standard molecular biology procedures as previously described (Da Cunha et al., 2017; Pais et al., 2019). The backbone plasmids used in this work included p2TK2-SW2 (Agaisse and Derré, 2013), a cloning vector suitable for transformation of *C. trachomatis*, and its derivative pVector[Pgp4^+^] (Da Cunha et al., 2017) (**Supplementary Table S1**), which enables the expression of proteins with a C-terminal double-hemagglutinin (2HA) tag in *C. trachomatis*. The accuracy of the nucleotide sequence of all the inserts or plasmids was confirmed by Sanger sequencing. In case of plasmid pVector[Pgp4^-^] with *pgp4* deleted (**Supplementary Table S1**), the accuracy of the nucleotide sequence of the entire plasmid was confirmed.

### *Escherichia coli* Strains and Growth Conditions

*Escherichia coli* NEB^®^ 10-beta (New England Biolabs) was used for plasmid construction and purification, and *E. coli* ER2925 (New England Biolabs) was used to replicate and purify plasmids for transformation of *C. trachomatis*. *E. coli* strains were grown in liquid or agar lysogeny broth (LB) with the appropriate selective antibodies and supplements. *E. coli* cells were transformed with the plasmids by electroporation.

### *C. trachomatis* Strains and Their Propagation and Transformation

The *C. trachomatis* strains used and generated in this work are listed in **Table 1**. They were propagated in HeLa 229 cells using standard procedures (Scidmore, 2005). Transformation of *C. trachomatis* was performed essentially as described by Agaisse and Derré (Agaisse and Derré, 2013), and in our previous studies (Da Cunha et al., 2017; Pais et al., 2019). Strains were purified by plaque assay using Vero cells, as described elsewhere (Nguyen and Valdivia, 2013). *Chlamydia* stocks were tested for *Mycoplasma* by conventional PCR (Uphoff and Drexler, 2011) and Sanger sequencing techniques. All newly generated *C. trachomatis* strains were checked for the presence of the desired plasmid by PCR using specific primers.

**Table 1:** *C. trachomatis* strains used and constructed in this study.

Strains	Description	Relevant genotype	Source/Refs.
L2/434/Bu ACE051	Wild-type strain.	*cteG^+^ pgp4^+^*	From Derek J. Fisher (originating from Tony Maurelli’s lab; University of Florida).
L2/434 (pVector[Pgp4^+^])	Derivative of L2/434/Bu ACE051carrying plasmid pVector[Pgp4^+^].	*cteG^+^ pgp4^+^*	This work.
L2/434 (pVector[Pgp4^-^])	Derivative of L2/434/Bu ACE051 carrying plasmid pVector[Pgp4^-^].	*cteG^+^ pgp4^-^*	This work.
*cteG::aadA*	Derivative of L2/434/Bu ACE051 with *cteG* inactivated.	*cteG^-^ pgp4^+^*	(Pais et al., 2019).
*cteG::aadA* (pCteG)	Derivative of *cteG::aadA* carrying plasmid pCteG.	*cteG^-^*/*cteG^+^ pgp4^+^*	This work.
*cteG::aadA* (pFabI-CteG)	Derivative of *cteG::aadA* carrying plasmid pFabI-CteG (harboring *cteG*, and *ctl0359/fabI*).	*cteG^-^*/*cteG^+^ pgp4^+^*	This work.
*cteG::aadA* (pFabI-CteG-CTL0361)	Derivative of *cteG::aadA* carrying plasmid pFabI-CteG-CTL0361 (harboring *cteG*, *ctl0359/fabI*, and *ctl0361*).	*cteG^-^*/*cteG^+^ pgp4^+^*	This work.
*cteG::aadA* (pVector[Pgp4^+^])	Derivative of *cteG::aadA* carrying plasmid pVector[Pgp4^+^].	*cteG^-^ pgp4^+^*	This work.
*cteG::aadA* (pVector[Pgp4^-^])	Derivative of *cteG::aadA* carrying plasmid pVector[Pgp4^-^].	*cteG^-^ pgp4^-^*	This work.
*cteG::aadA* (pCteG-2HA/pCteG-2HA[Pgp4^+^])	Derivative of *cteG::aadA* carrying plasmid pCteG-2HA/pCteG-2HA[Pgp4^+^].	*cteG^-^*/*cteG-2HA^+^ pgp4^+^*	(Pais et al., 2019).
*cteG::aadA* (pCteG-2HA[Pgp4^-^])	Derivative of *cteG::aadA* carrying plasmid pCteG-2HA[Pgp4^-^].	*cteG^-^*/*cteG-2HA^+^ pgp4^-^*	This work.
L2/25667R	Plasmidless L2 strain.	*cteG^+^ pgp4^-^*	(From Agathe Subtil; Peterson et al., 1990).

### Infection of HeLa Cells With *C. trachomatis*

Infections for quantification of inclusion forming units (IFUs), cell cytotoxicity assays, determination of inclusion size and assessment of protein levels by immunoblotting were carried out by seeding 1×10^5^ HeLa cells per well in 24-well plates. For immunofluorescence experiments, cells were seeded onto 13 mm glass coverslips. The day after seeding, cells were infected with *Chlamydia* inocula at various multiplicities of infection (MOIs) and periods of time, as previously described (Da Cunha et al., 2017). To determine the effect of the addition of gentamicin in the number of recovered IFUs or in cytotoxicity levels, media supplemented with 10 µg/mL of gentamicin was added to cells at 0 h of infection. At 24 h post-infection, cells were washed once with DMEM supplemented with 10% (v/v) FBS and left in fresh media without gentamicin for the remainder time of infection. For determination of inclusion size, cells were infected at a MOI of 0.06 for 24 h before fixation. To assess protein levels by immunoblotting, cells were infected with a MOI of 6 and incubated for 16, 24, 30 or 40 h in DMEM supplemented with 10% (w/v) FBS and 10 µg/mL of gentamicin.

For detection by immunoblotting of bacteria in the supernatant of infected cells, 5×10^5^ HeLa cells per well were seeded in 6-well plates. Cells were infected as described above with a MOI of 0.06 and incubated at 37°C for 38 h in DMEM supplemented with 10% (v/v) FBS. At this time, the medium was replaced by DMEM without FBS, and the infection allowed to proceed up to 48 h.

### Assessment of Relative Progeny Generation and Quantification of IFUs in Infected Cells

Assessment of progeny generation was performed essentially as previously described (Sixt et al., 2017). Briefly, two identical 24-well plates seeded with HeLa cells were infected with *C. trachomatis* strains at a MOI of 0.06. In one of the plates, infected cells were fixed at 24 h post-infection with methanol for 7 min at -20°C (input). In the other plate, infection was allowed to proceed for 40 h, cells were washed very briefly with H_2_O and then osmotically lysed by incubation for 15 min at room temperature with 500 µL of H_2_O. The lysed cells were vigorously resuspended by pipetting up and down several times and the suspension was added to 500 µL of double concentrated sucrose phosphate glutamate buffer (2x SPG; 0.44 M sucrose, 34 mM Na_2_HPO_4_, 6 mM NaH_2_PO_4_, 10 mM L-glutamic acid). The lysates obtained were homogenized by vortexing, serial diluted in SPG (0.22 M sucrose, 17 mM Na_2_HPO_4_, 3 mM NaH_2_PO_4_, 5 mM L-glutamic acid), and used to infect a fresh layer of HeLa cells. These cells were fixed with methanol 24 h post-infection for 7 min at -20°C (output), and immunolabelled. Inclusions were counted by fluorescence microscopy in ≥30 fields of duplicated samples, using a total amplification of 400x, and IFUs/mL were determined as previously described (Scidmore, 2005). For each strain, the relative progeny generation was obtained by dividing the number of IFUs in the output by those in the input.

For quantification of IFUs in the cell culture supernatant and cell lysate fractions of infected cells, the supernatants (1 mL) were collected and vortexed to homogenize extracellular bacteria (supernatant fraction). Attached cells were washed once with H_2_O and lysed by osmotic shock, as described above for assessment of progeny generation. Lysed cells were re-suspended and vortexed to homogenize recovered intracellular bacteria (lysate fraction). Both fractions were serial diluted in SPG and the quantification of IFUs was done as for assessment of progeny generation.

### Cell Cytotoxicity Assays

The supernatants of infected HeLa cells were assayed for released lactate dehydrogenase (LDH) with the CytoScan™ LDH Cytotoxicity Assay kit (G-Biosciences), following the manufacturer’s instructions and including the appropriate controls. To calculate the % of LDH released, in each assay and time-point the amount of LDH activity detected in uninfected cells after lysis with 1% (v/v) Triton X-100, and the amount of LDH activity released from uninfected cells, were determined. The % of LDH released was then calculated as 100 x [(LDH activity released from infected cells - LDH activity released from uninfected cells)/(LDH activity detected in uninfected cells after lysis with Triton X-100 - LDH activity released from uninfected cells)]. Absorbance at 490 nm was measured in a SpectraMax 190 microplate reader (Molecular Devices) and data was acquired using the SoftMax Pro 7.1 software (Molecular Devices).

### Antibodies and Dyes

For immunoblotting, the following primary antibodies were used: rat monoclonal anti-HA (3F10, Roche, diluted 1:1,000), mouse monoclonal anti-chlamydial Hsp60 (A57-B9; Thermo Fisher Scientific, 1:1,000), mouse monoclonal anti-α-tubulin (clone B-5-1-2, Sigma Aldrich, 1:1,000). Anti-mouse or anti-rat secondary antibodies were all horseradish peroxidase (HRP)-conjugated (GE Healthcare and Jackson ImmunoResearch, 1:10,000).

For immunofluorescence microscopy, the following primary antibodies were used: goat polyclonal anti-*Chlamydia* major outer membrane protein (MOMP) (Abcam, 1:200), rat monoclonal anti-HA (3F10, Roche, 1:200), rabbit polyclonal anti-GM130 (Sigma Aldrich, 1:200), mouse monoclonal anti-TGN46 (clone TGN46-8, Sigma Aldrich, 1:200) and goat anti-*C. trachomatis* FITC-conjugated polyclonal antibody (Sigma-Aldrich, 1:150). The secondary antibodies were all purchased from Jackson ImmunoResearch and diluted 1:200: Rhodamine Red-X-conjugated anti-rat, AF568-conjugated anti-mouse, DyLight 405-conjugated anti-goat; Cyanine 3 (Cy3)-conjugated anti-rabbit. DAPI (4′,6-Diamidino-2-phenylindole; 1:30.000) was used to label DNA, and actin staining was carried out by incubating HeLa cells with Phalloidin-Alexa 488 (Thermo Fisher Scientific, 1:100).

### Immunoblotting

For immunobloting, cells were harvested by trypsinization and washed as previously described (Da Cunha et al., 2017; Pais et al., 2019), and immediately re-suspended and boiled in SDS-PAGE Laemmli buffer (SDS loading buffer). For the analysis of supernatants of infected cells, SDS loading buffer was added immediately and directly to the supernatants. All samples were boiled for 5 minutes at 100°C. and then separated by 12% (v/v) SDS-PAGE and transferred onto 0.2 μm nitrocellulose membranes (Bio-Rad) using Trans-Blot Turbo Transfer System (BioRad). Detection was done with SuperSignal West Pico Chemiluminescent Substrate (Thermo Fisher Scientific) or SuperSignal West Femto Maximum Sensitivity Substrate (Thermo Fisher Scientific), as specified in figure legends, and exposure to Amersham Hyperfilm ECL (GE Healthcare) as previously described (Da Cunha et al., 2017; Pais et al., 2019).

### Immunofluorescence Microscopy

Infected HeLa cells were fixed either with freezing methanol (-20°C) for 7 min or with 4% (w/v) paraformaldehyde (PFA) for 15 min at room temperature, as specified in figure legends. For immunolabelling, antibodies were diluted in phosphate buffered saline (PBS) containing 10% (v/v) horse serum. When cells where fixed with PFA, 0.1% (w/v) saponin was added to allow cell permeabilization. All incubations were done for 1 h at room temperature. Cells were washed with PBS or PBS containing saponin between incubation with each antibody, and finally in PBS and H_2_O. The coverslips were assembled on microscopy glass slides using Aqua-Poly/Mount (Polysciences) mounting medium and cells were examined by fluorescence microscopy in a Axio Imager.D2 (Zeiss) upright microscope. Images were collected by an Axiocam MRm (Zeiss) camera and processed with Zeiss ZEN (Zeiss) software, Fiji software (Schindelin et al., 2012) and Adobe Illustrator.

### Statistical Analysis

All statistical analysis was performed with GraphPad Prism, version 9 for MacOS (GraphPad Software, San Diego, California, USA, https://www.graphpad.com). Statistic tests are specified in the legend of each figure. When necessary, data for statistical analysis was transformed by applying the natural logarithm, which rendered the distribution of populations Gaussian. Statistical differences were considered significant when p < 0.05.

### Whole-Genome Sequencing and Bioinformatics

*C. trachomatis* L2/434 and its derivative *cteG::aadA* (Pais et al., 2019) (**Table 1**) were subjected to whole-genome sequencing (WGS). For this, an optimized DNA purification procedure was used to ensure depletion of human nucleic acids. First, suspensions of infected HeLa 229 cells were sonicated (3x10s, 50%, 5 K cycles/s; VibraCell, Bioblock Scientific) and the cell debris were discarded through low-speed centrifugation. Subsequently, the chlamydiae in the supernatant were pelleted by high-speed centrifugation, followed by resuspension in a DNase/RNase cocktail [stock solution with 4.6 mg/ml DNase (Sigma; 400 Kunitz U/mg) and 13 mg/ml RNase (Applichem; 100.8 Kunitz U/mg), in Hanks’ Balanced Salt Solution (HBSS), diluted 1:10 in HBSS], sonication (2x20s; S30 Elmasonic), and incubation at 37°C for 20 min. The DNase and RNase were then inactivated by incubation at 65°C for 15 min, followed by chilling 1 min on ice. The chlamydiae in the suspensions were again pelleted, resuspended in PBS, and then added over a layer of 30% (v/v) urographin [diluted from 76% (v/v) urographin, sodium amidotrizoate (0.1 g/ml) and meglumine amidotrizoate (0.66 g/ml); Bayer, Portugal)]. A high-speed centrifugation step was then carried out and the chlamydiae-enriched fraction was collected, resuspended in PBS, and subjected to a second round of DNase/RNase digestion and inactivation. The chlamydiae in these suspensions were then pelleted, washed with PBS, and further processed for DNA isolation using Proteinase K (20 mg/mL) lysis and the QIAamp DNA Mini Kit (Qiagen, Valencia, CA, USA), according to manufacturer’s instructions. Purified DNA was subsequently subjected to Nextera XT library preparation and subsequent paired-end sequencing (2x250 bp) in Illumina MiSeq (Illumina Inc., San Diego, CA, USA), according to the manufacturer’s instructions. Bioinformatics analysis involved: i) reads’ quality analysis and cleaning/improvement using FastQC (https://www.bioinformatics.babraham.ac.uk/projects/fastqc/) and Trimmomatic (http://www.usadellab.org/cms/?page=trimmomatic) (Bolger et al., 2014); ii) reference-based mapping and SNP/indel analysis against the *C. trachomatis* L2/434/Bu reference genome sequences (NCBI accession numbers: AM884176.1 for chromosome and AM886278.1/X07547.1 for the plasmid) using Snippy v 3.2 (https://github.com/tseemann/snippy); iii) *de novo* genome assembly using SPAdes v 3.11.0 (Bankevich et al., 2012); iv) whole genome alignment and inspection using Mauve v 2.3.1 (Darling et al., 2010); and v) SNP/indel inspection using the Integrative Genomics Viewer (http://www.broadinstitute.org/igv) (Robinson et al., 2011). This procedure for *C. trachomatis* enrichment allowed obtaining a percentage of *“on-target”* reads above 99.5% for both strains. WGS raw reads were submitted to the European Nucleotide Archive (ENA) under the BioProject accession number PRJEB51643.

## Results

### A *C. trachomatis cteG::aadA* Insertional Mutant Has a Defect in Progeny Generation

**Figure 1:**
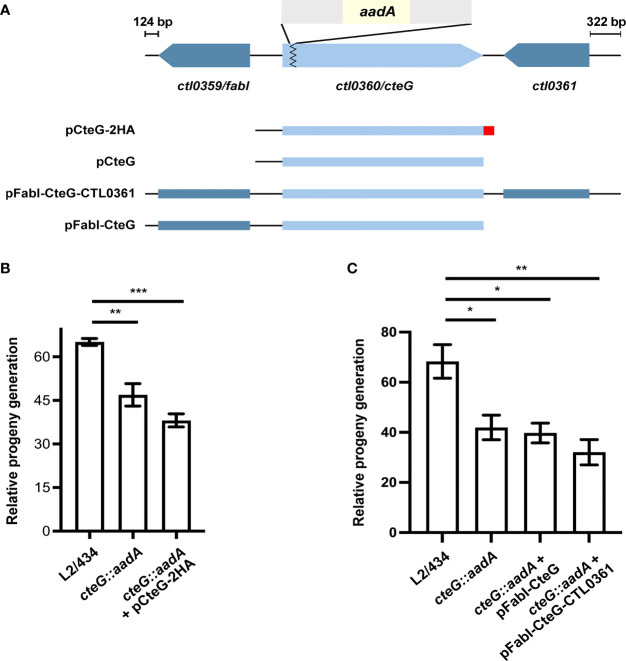
A *C. trachomatis cteG::aadA* insertional mutant is defective in progeny generation. **(A)** Schematic representation of the genomic region of *ctl0360*/*cteG* (light blue), which was disrupted by insertion of a modified group II intron (grey) carrying a spectinomycin-resistance gene, *aadA* (yellow) to generate a *C. trachomatis cteG* mutant strain (*cteG::aadA*) (Pais et al., 2019). The *cteG::aadA* mutant strain was transformed with plasmids encoding CteG fused to a double hemagglutinin tag (2HA; red; pCteG-2HA; also named pCteG-2HA[Pgp4^+^] in **Table 1** and in **Figures 4**, **5**), native CteG (pCteG) (**Supplementary Figure S1**), or CteG and two (*ctl0359/fabI* and *ctl0361*; pFabI-CteG-CTL0361) or one (*ctl0359/fabI*; pFabI-CteG) of its flanking genes (dark blue). **(B)** Two identical tissue culture plates seeded with HeLa cells were infected with *C. trachomatis* L2/434, *cteG::aadA* mutant and complemented (*cteG::aadA* harboring a plasmid encoding CteG-2HA, also named pCteG-2HA[Pgp4^+^] in **Table 1** and in **Figures 4**, **5**) strains at a MOI of 0.06. In one plate (input), the IFUs obtained in a primary infection were quantified at 24 h p.i. by immunofluorescence microscopy after fixation and immunolabelling of the chlamydiae; in the second plate (output), cells were lysed at 40 h p.i. and the number of released infectious particles were quantified after infecting for 24 h a new plate seeded with HeLa cells followed by fixation, immunolabelling of the chlamydiae, and immunofluorescence microscopy. For each strain, the relative progeny generation was obtained by dividing the number of IFUs in the output by those in the input. See Materials and Methods for a detailed description of the procedure. **(C)** *cteG::aadA* mutant strains harboring pFabI-CteG or pFabI-CteG-CTL0361 (see Panel **A**) were assessed in terms of infectious progeny generation as in **(B)** by comparison with the parental (L2/434) and mutant (*cteG::aadA*) strains. Data in **(B, C)** correspond to the mean ± standard error of the mean (n=3). Statistical significance was determined by using ordinary one-way ANOVA and Dunnett post-test analysis relative to the L2/434 strain (*p<0.05; **p<0.01; ***p<0.001).

In a previous report, we observed that a *C. trachomatis cteG::aadA* mutant strain, generated with a modified group II intron containing a spectinomycin-resistance gene (*aadA*) (**Figure 1A**), produces smaller inclusions comparing to its parental L2/434 strain (Pais et al., 2019). This could not be complemented by CteG with a C-terminal double hemagglutinin tag (CteG-2HA) encoded in a plasmid, with the gene expressed from the endogenous *cteG* promoter (Pais et al., 2019). However, in this previous work, we did not observe significant differences in the generation of infectious progeny of the mutant and complemented strains relative to the L2/434 strain (Pais et al., 2019). To clarify these issues, we started by reassessing the generation of infectious progeny, but also quantifying in each assay the number of internalized IFUs for each strain. This revealed a slight (~1.5-fold), but significant, difference between the L2/434 parental strain and the mutant and complemented strains (**Figure 1B**). To address the reason for lack of complementation, a possible interference of the 2HA tag on the activity of CteG was excluded, as a *C. trachomatis cteG::aadA* strain encoding native CteG in a plasmid also produced smaller inclusions comparing to the parental strain (**Figure 1A** and **Supplementary Figure S1A**). Furthermore, *C. trachomatis cteG::aadA* strains harboring plasmids encoding *cteG* and one (*ctl0359*/*fabI*) or both (*ctl0359*/*fabI* and *ctl0361*) of its flanking genes (**Figure 1A**) did not complement the defects in progeny generation (**Figure 1C**) or in inclusion area (**Supplementary Figure S1B**). This excluded a polar effect in *ctl0359*/*fabI* or *ctl0361* (**Figure 1A**) arising from the insertion of the intron within *cteG.* Finally, WGS of the parental and mutant strains revealed four nucleotide changes in the *cteG::aadA* strain that led to missense mutations, for example in an Inc (CT618) and in a T3S gene (LcrH/CT862), as well as two nucleotide changes in non-coding regions (**Supplementary Table S3**). Therefore, the observed differences in inclusion size and progeny generation between the wild-type strain and the *cteG::aadA* mutant strain are not due to the disruption of *cteG*, or to an indirect effect of the disruption on neighboring genes, but possibly because of at least one of the nucleotide changes detected in the *cteG::aadA* mutant strain. However, within the scope of this work we did not study how the identified mutations may result in the observed defects.

### The *cteG::aadA* Mutant Strain Shows a CteG-Dependent Defect in Egress From Infected Host Cells

**Figure 2:**
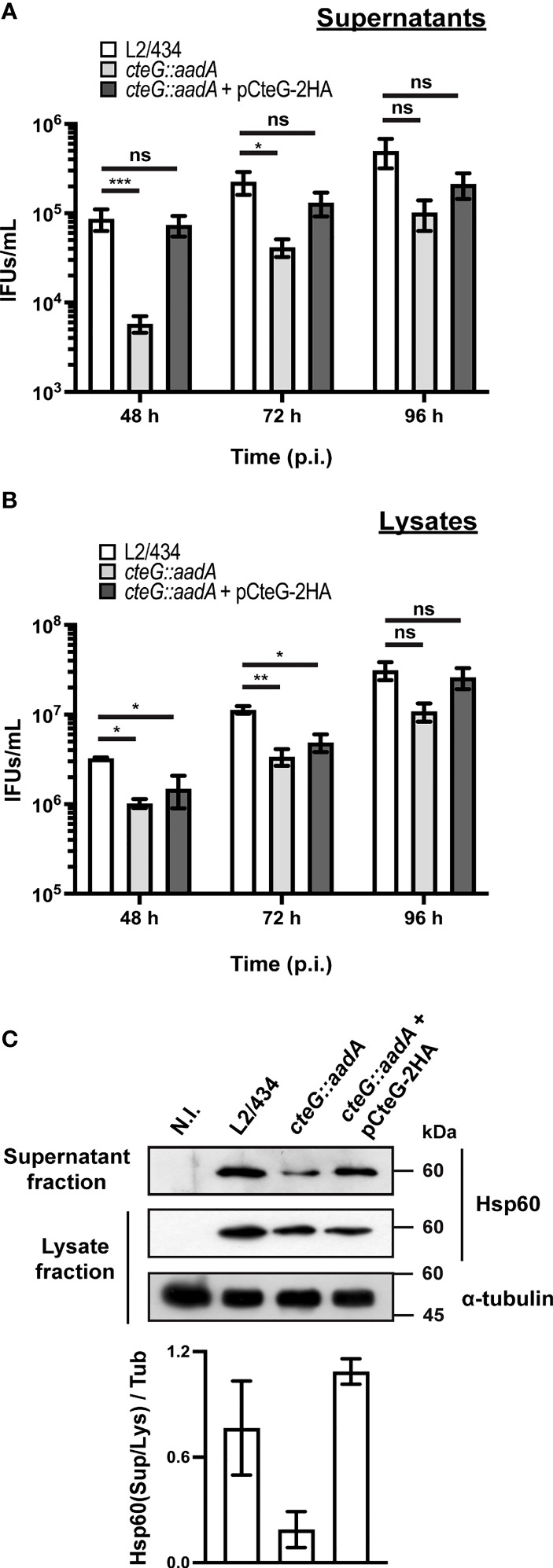
*C. trachomatis* displays a CteG-dependent defect in egress from infected host cells. HeLa 229 cells were infected with *C. trachomatis* parental (L2/434), mutant (*cteG::aadA*), and complemented (*cteG::aadA* harboring a plasmid encoding CteG-2HA; also named pCteG-2HA[Pgp4^+^] in **Table 1** and in **Figures 4**, **5**) strains at an MOI of 0.06 for 48, 72 or 96 h. At each time post-infection (p.i.), cell supernatants were collected (supernatant fraction) and adherent cells were lysed by osmotic shock to recover intracellular chlamydiae (lysate fraction). Fresh layers of HeLa cells were infected with serial dilutions of both supernatant **(A)** and lysate **(B)** fractions to quantify the number of recoverable inclusion-forming units (IFUs/mL). Data correspond to the mean ± standard error of the mean (n≥3). For each time point, statistical significance was determined by using ordinary one-way ANOVA and Dunnett post-test analysis relative to the L2/434 parental strain (ns, non-significant; *p<0.5; **p<0.01, ***p<0.001). For statistical analysis, natural logarithm was applied to data to ensure normality of the populations. **(C)** HeLa cells were left non-infected (N.I.), or were infected for 48 h with *C. trachomatis* L2/434, *cteG::aadA* or *cteG::aadA* harboring pCteG-2HA (also named pCteG-2HA[Pgp4^+^] in **Table 1** and in **Figures 4**, **5**) at a MOI of 0.06. The proteins in the supernatant fraction (containing extracellular bacteria) were analyzed by immunoblotting with an antibody against *C. trachomatis* Hsp60 and the lysate fraction (intracellular bacteria) was analyzed by immunoblotting with antibodies against *C. trachomatis* Hsp60 and human α-tubulin (cell loading control), and using SuperSignal West Pico detection kit (Thermo Fisher Scientific) to detect proteins in the lysate fraction or SuperSignal West Femto detection kit (Thermo Fisher Scientific) to detect proteins in the supernatant fraction. Bands were quantified using Fiji software, and the Hsp60 signal in the supernatant fraction (Sup) was normalized to that in the lysate fraction (Lys) and to tubulin signal (Tub). Bars correspond to mean ± standard error of the mean (n=3).

**Figure 3:**
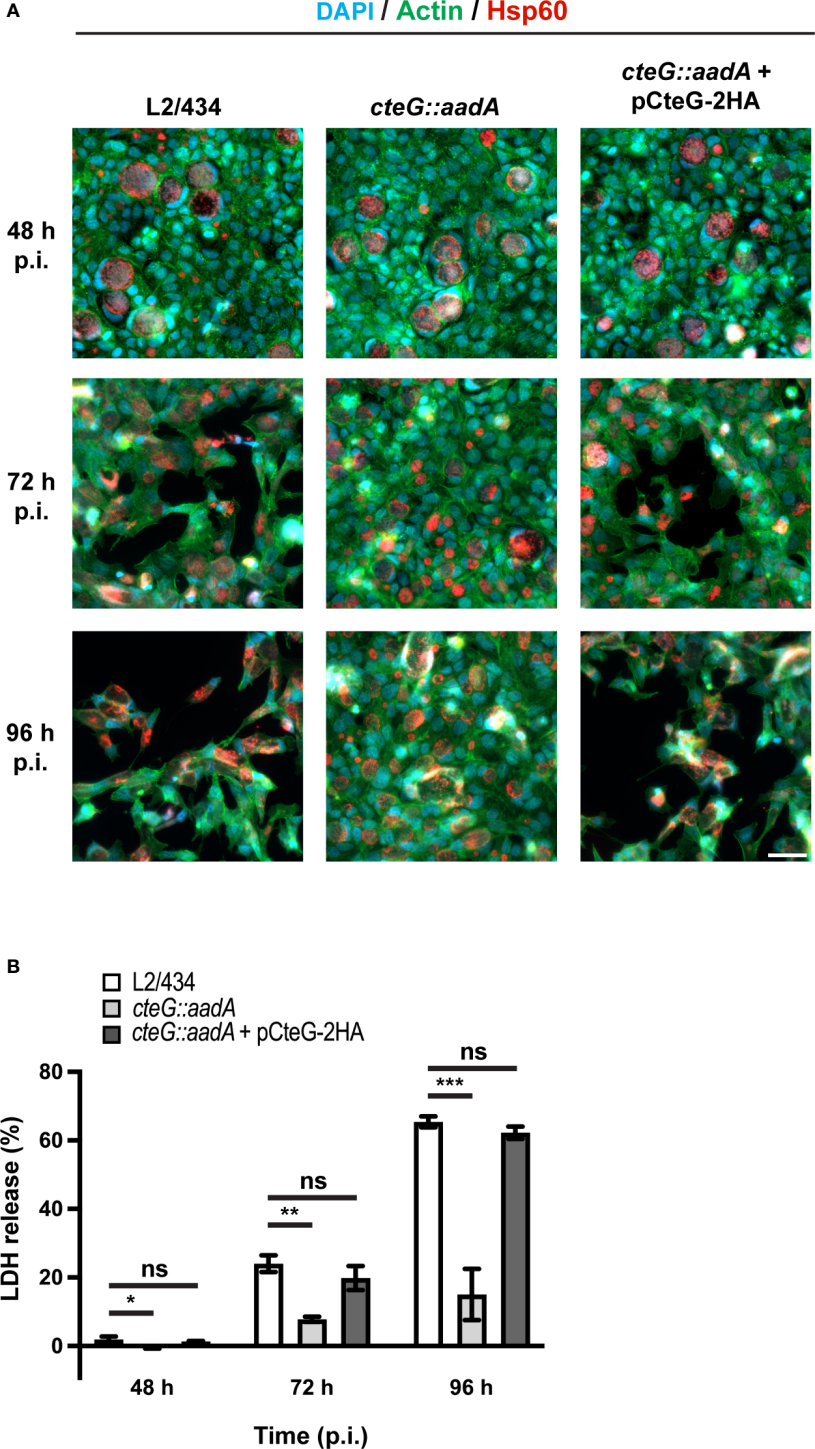
*C. trachomatis* displays a CteG-dependent defect in host cell lysis. HeLa 229 cells were infected with *C. trachomatis* parental (L2/434), mutant (*cteG::aadA*), and complemented (*cteG::aadA* harboring a plasmid encoding CteG-2HA; also named pCteG-2HA[Pgp4^+^] in **Table 1** and in **Figures 4**, **5**) strains at a MOI of 0.3. **(A)** At 48, 72 or 96 h post-infection (p.i.), cells were fixed with methanol, immunolabelled with antibodies against *C. trachomatis* Hsp60 (red) and appropriate fluorophore-conjugated secondary antibodies, and stained with DAPI (host cell nuclei and chlamydial inclusions; blue) and with fluorophore-conjugated phalloidin (host actin cytoskeleton; green). Scale bar, 40 µm. **(B)** At 48, 72, and 96 h p.i., the release of host lactate dehydrogenase (LDH) into the supernatant of infected HeLa cells was measured using a CytoScan™ LDH Cytotoxicity Assay kit (G-Biosciences). Data are representative of five independent experiments and correspond to the mean ± standard error of the mean of three biological replicates. For each time point, statistical significance was determined by using ordinary one-way ANOVA and Dunnett post-test analysis relative to the L2/434 parental strain (ns, non-significant; *p<0.05; **p<0.01; ***p<0.001).

The localization of CteG at the host cell plasma membrane at later times of host cell infection led us to hypothesize that this effector could be involved in *C. trachomatis* egress. To analyze this, HeLa cells were infected with the *C. trachomatis* parental (L2/434), mutant (*cteG::aadA*), and complemented (*cteG::aadA* harboring a plasmid encoding CteG-2HA) strains for 48, 72 or 96 h at different multiplicities of infection (MOIs). The experiments were performed in the absence of gentamicin, to avoid possible killing of externalized chlamydiae. At each time point, tissue culture cell supernatants were collected (supernatant fraction). Adherent cells were subsequently lysed by osmotic shock, enabling recovery of chlamydiae that remained intracellular (lysate fraction). As shown in **Figure 2A**, with a MOI of 0.06, and at 48 and 72 h post-infection, significantly less IFUs were present in the supernatant fraction of cells infected with the *cteG* mutant strain comparing to the L2/434 strain. This phenotype was restored in the complemented strain (**Figure 2A**). Therefore, the presence of less *C. trachomatis* infectious particles in the cell culture supernatant of cells by the *cteG::aadA* mutant strain is CteG-dependent and is not related to the defect in progeny generation of the mutant as this latter defect is also displayed by the complemented strain (**Figures 1**, **2B**). Furthermore, the calculated ratios between the number of IFUs in the supernatant (**Figure 2A**) against the total IFUs (supernatant and lysates; **Figures 2A, B**) at 48 h post-infection were 2.6 ± 0.6% (mean±SEM) for the L2/434 strain, 6.0 ± 1.6% for the complemented strain, while only 0.6 ± 0.1% for the *cteG::aadA* mutant strain. This CteG-dependent phenotype was also observed at higher MOIs (0.3, 1.5, or 3; **Supplementary Figure S2A**), and regardless of the presence or absence of gentamicin in the culture medium between 0 and 24 h post-infection (**Supplementary Figure S3A**). At 96 h post-infection the phenotype was less obvious (**Figure 2A**; **Supplementary Figure S2A**), in part possibly because of re-infection events that interfere with the quantification of IFUs in the culture supernatants. In addition, at 72 and 96 h post-infection, at a higher MOI of 3, there were significantly more IFUs in the lysates of cells infected by the *cteG::aadA* mutant strain than in cells infected by the parental or complemented strains (**Supplementary Figure S2B**), as a consequence of the progressive destruction of the cell monolayer in cells infected by chlamydiae producing CteG (see **Figure 3A** below). This gradual reduction in viable host cells might also interfere with measurements of IFUs in the supernatant of cells infected at higher MOIs and for longer times. Finally, analyzing the proteins in the supernatant and lysate fractions by immunoblotting with an anti-*C. trachomatis* Hsp60 antibody confirmed that the culture supernatant of cells infected by the *C. trachomatis cteG::aadA* mutant contains less chlamydiae relative to cells infected by the L2/434 strain, and that this is CteG-dependent (**Figure 2C**). Overall, this indicated that CteG is involved in *C. trachomatis* egress from host cells, likely by contributing to host cell lysis.

### The *cteG::aadA* Mutant Strain Shows a CteG-Dependent Defect in Host Cells Lysis

As mentioned above, preliminary phase-contrast microscopy observations indicated that the significantly higher amounts of recoverable IFUs in lysates of HeLa cells infected with a MOI of 3 for 72 or 96 h by the *cteG::aadA* mutant relative to the parental L2/434 and complemented strains (**Supplementary Figure S2B**) were a direct consequence of a much more pronounced destruction of the HeLa cell monolayer by the parental and complemented strains. To visualize this directly, we analyzed cells infected (MOI of 0.3) for 48, 72 or 96 h with the parental, mutant, and complemented strains by immunofluorescence microscopy. Infected cells were fixed and stained with an anti-chlamydial Hsp60 antibody (to visualize chlamydial inclusions), fluorophore-conjugated phalloidin (to visualize the host actin cytoskeleton) and with DAPI (to visualize the host cell nuclei). The representative images on **Figure 3A** illustrate that the monolayer of HeLa cells infected with the mutant strain remained relatively intact, even at 96 h post-infection, whereas the monolayer of cells infected with either the parental or the complemented strain was visibly destroyed from 72 h post-infection.

To perform a direct measurement of host cell lysis, we monitored the release of LDH into the supernatant of HeLa cells infected at different MOIs, for 48, 72, and 96 h, by the parental, mutant, and complemented strains. As illustrated in **Figure 3B**, at a MOI of 0.3, the *cteG::aadA* mutant strain showed a lower ability to cause host cell lysis by comparison with the parental and complemented strains. Similar observations were made at lower (0.06) and higher MOIs (1.5 or 3) (**Supplementary Figure S4**), and regardless of the presence of gentamicin in the cell culture media from 0 to 24 h post-infection (**Supplementary Figure S3B**). While part of the chlamydiae that we detected in the culture supernatant of infected cells (**Figure 2**) could be released by extrusion, altogether, these data indicated that, from ~48 h post-infection of HeLa cells, CteG promotes chlamydial egress by contributing to host cell lysis by *C. trachomatis*.

### Production and Localization of CteG Are Not Regulated by *C. trachomatis* Virulence Plasmid Encoded Pgp4

We confirmed previous observations that the virulence plasmid contributes to *C. trachomatis* lytic exit (Yang et al., 2015) by infecting HeLa cells with the L2/434 strain side by side with a plasmidless *C. trachomatis* strain (25667R) and monitoring LDH release at 48, 72, and 96 h post-infection (**Supplementary Figure S5**). The plasmid-dependent role on host cell lysis has been shown to be due to plasmid-encoded Pgp4 (Yang et al., 2015), which mediates transcriptional regulation of several plasmid and chromosomal genes (Carlson et al., 2008; Song et al., 2013). However, expression of *cteG* is not regulated by Pgp4 (Song et al., 2013; Patton et al., 2018).

To study how CteG and Pgp4 contribute to chlamydial lytic exit, we generated several *C. trachomatis* strains harboring recombinant plasmids carrying (Pgp4^+^) or lacking (Pgp4^-^) the *pgp4* gene (**Table 1**). It has been previously shown that during chlamydial transformation the *C. trachomatis* native plasmid is eventually lost by exchange with the novel plasmid (Wang et al., 2011; Mueller et al., 2016). To ensure that the newly generated Pgp4^+^ or Pgp4^-^ *C. trachomatis* strains lost the native plasmid, we verified both the presence of the desired recombinant plasmid (**Supplementary Figure S6**) and the loss of the native plasmid (**Supplementary Figure S7**).

**Figure 4:**
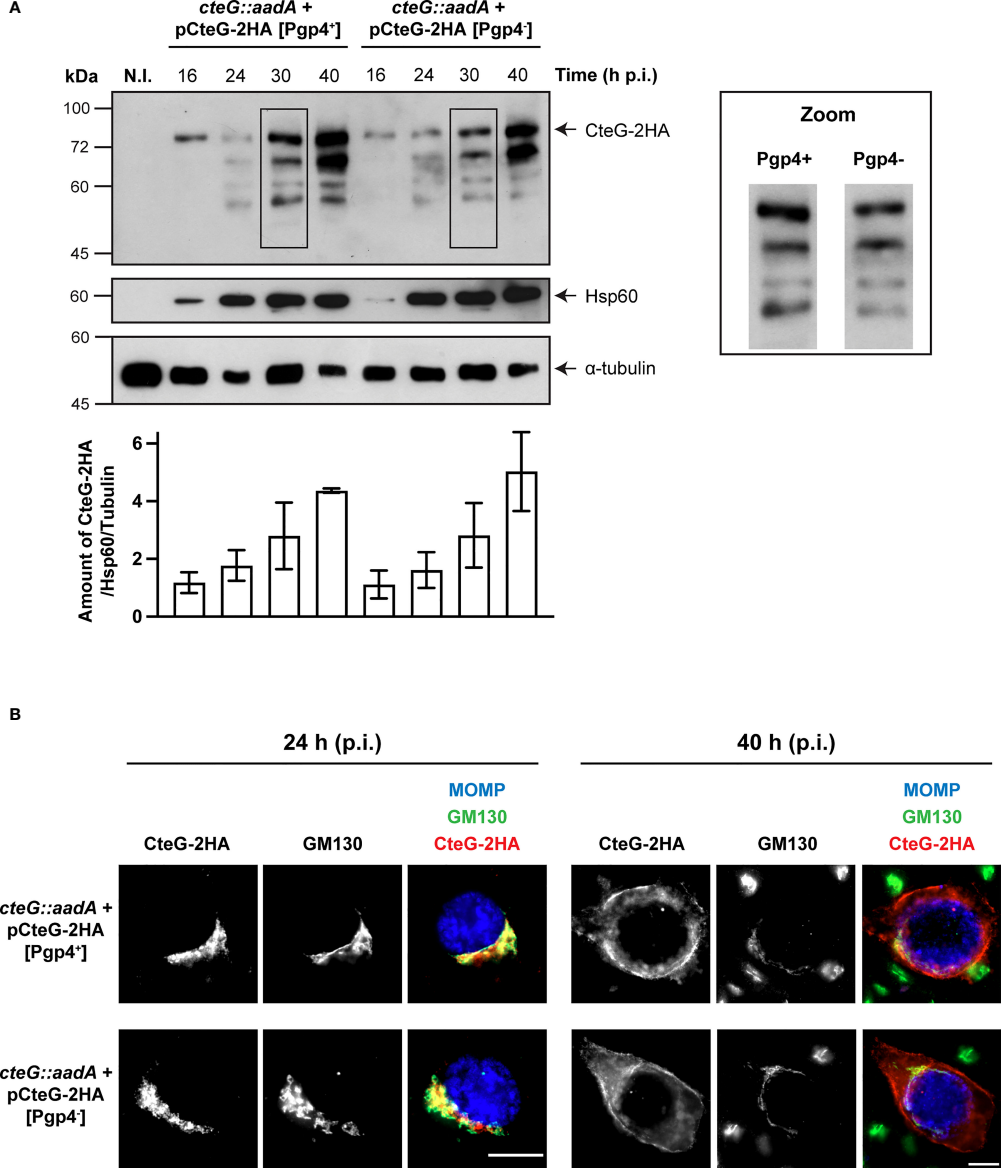
Pgp4 does not modulate the production or the localization of CteG during *C. trachomatis* infection. HeLa cells were either left non-infected (N.I.) or infected with *C. trachomatis cteG::aadA* strains carrying pCteG-2HA[Pgp4^+^] (also named pCteG-2HA in **Table 1** and in **Figures 1**–**3**; Pgp4^+^/CteG-2HA^+^) or pCteG-2HA[Pgp4^-^] (Pgp4^-^/CteG-2HA^+^) **(A)** At 16, 24, 30 or 40 h post-infection (p.i.), whole cell extracts were prepared and then analyzed by immunoblotting with antibodies against HA (CteG-2HA), *C. trachomatis* Hsp60 (bacterial loading control) and human α-tubulin (HeLa cell loading control), and using SuperSignal West Pico detection kit (Thermo Fisher Scientific) to detect Hsp60 or α-tubulin, or SuperSignal West Femto detection kit (Thermo Fisher Scientific) to detect CteG-2HA. The band corresponding to full-length CteG-2HA is indicated with an arrow. Zooms of the band pattern of CteG-2HA species at 30 h p.i. in both *pgp4^+^* and *pgp4^-^* backgrounds are displayed. The intensity of all bands on each lane was quantified using the software Fiji and summed to obtain the intensity of all CteG-2HA species at a given time point. Each value was normalized to the bacterial and HeLa cell loading controls. Bars correspond to mean ± standard error of the mean (n=3). **(B)** Cells were fixed with PFA 4% (w/v) at 24 or 40 h p.i. and immunolabelled with antibodies against *C. trachomatis* major outer membrane protein (MOMP; blue), *cis*-Golgi network (GM130; green) and HA (CteG-2HA; red), and appropriate fluorophore-conjugated secondary antibodies. Scale bar, 10 µm.

First, to analyze if Pgp4 influences the production or the subcellular localization of CteG, HeLa cells were infected for 16, 24, 30 and 40 h with *C. trachomatis cteG::aadA* strains encoding CteG-2HA on a plasmid, but in one case with a plasmid carrying Pgp4 (pCteG-2HA[Pgp4^+^]; Pais et al., 2019) and in the other not (pCteG-2HA[Pgp4^-^]; **Table 1**), followed by immunoblotting of whole cell extracts. In these two plasmids the expression of the hybrid *cteG-2HA* gene is under the control of the *cteG* promoter, mimicking endogenous regulation. Quantitative analysis of immunoblots revealed that Pgp4 does not regulate the timing or amount of CteG-2HA production (**Figure 4A**). Previously, we reported the appearance of faster migrating species of CteG-2HA detected by immunoblotting with the anti-HA antibody of extracts of HeLa cells infected with *C. trachomatis* producing CteG-2HA for more than 20-24 h (Pais et al., 2019). These faster migrating species are indicative of CteG degradation or processing occurring within the chlamydiae (Pais et al., 2019). It is currently unknown if they have functional relevance or are a consequence of plasmid-mediated overexpression of CteG-2HA, but Pgp4 does not influence their appearance (**Figure 4A**). Finally, by immunofluorescence microscopy of cells infected by these two strains, and subsequent quantitative analysis of the localization of CteG-2HA, we showed that CteG-2HA localizes at the Golgi (at 24 h post-infection) or at the host cell plasma membrane (at 40 h post-infection) during infection of host cells by *C. trachomatis* regardless of the presence or absence of Pgp4 (**Figure 4B** and **Supplementary Figure S8A**). We found only minor and no significant differences in Golgi distribution around the inclusion when analyzing cells infected by the *cteG::aadA*(pCteG-2HA[Pgp4^+^]) or *cteG::aadA*(pCteG-2HA[Pgp4^-^]) strains by immunofluorescence microscopy (**Supplementary Figures S8B, C**). Therefore, and in summary, Pgp4 does not control the production or localization of CteG.

### A *cteG::aadA pgp4* Double Mutant Strain Displays a Defect in Inducing Host Cell Lysis Identical to *cteG::aadA* or *pgp4* Single Mutants

**Figure 5:**
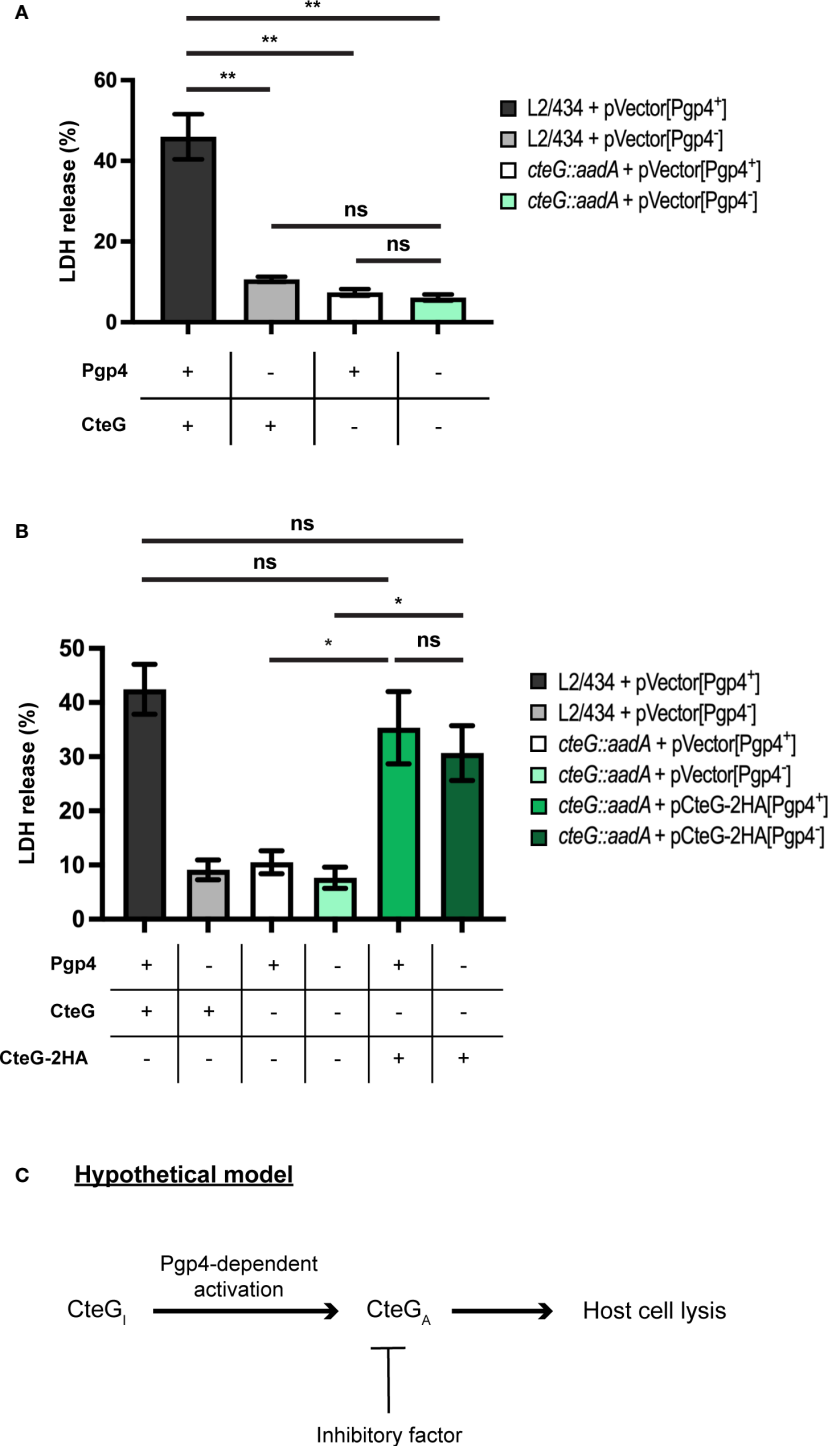
*C. trachomatis* mediates host cell lysis *via* a common pathway involving both CteG and Pgp4. **(A)** HeLa cells were infected with *C. trachomatis* L2/434 carrying pVector[Pgp4^+^] (CteG^+^/Pgp4^+^), L2/434 carrying pVector[Pgp4^-^] (CteG^+^/Pgp4^-^), *cteG::aadA* carrying pVector[Pgp4^+^] (CteG^-^/Pgp4^+^), or *cteG::aadA* carrying pVector[Pgp4^-^] (CteG^-^/Pgp4^-^) for 72 h at an MOI of 0.3, and the LDH released by lysed host cells was measured using a CytoScan™ LDH Cytotoxicity Assay kit (G-Biosciences). **(B)** As in panel A, but HeLa 229 cells were also infected with *cteG::aadA* carrying pCteG-2HA[Pgp4^+^] (also named pCteG-2HA in **Table 1** and in **Figures 1**–**3**; CteG-2HA^+^/Pgp4^+^), or *cteG::aadA* carrying pCteG-2HA[Pgp4^-^] (CteG-2HA^+^/Pgp4^-^). Statistical significance was assessed by using ordinary one-way ANOVA and Tukey post-test analysis. In **(A, B)**, data correspond to mean ± standard error of the mean (n = 4 in panel A and n = 7 in panel B; ns, non-significant; *p<0.01; **p<0.0001). **(C)** Hypothetical model for the mode of action of CteG and Pgp4 in promoting host cell lysis. After CteG is produced by chamydiae in an inactive form (CteG_I_), CteG is activated (CteG_A_) in a Pgp4-dependent manner, which could occur within chlamydiae or after delivery of CteG into the host cell cytoplasm. Premature CteG_A_-mediated host cell lysis is prevented by the action of an unknown inhibitory factor, which could be another chlamydial effector or a host cell factor. At late stages of infection, the effect of this inhibitor in CteG_A_ is alleviated by an unknown mechanism and host cell lysis is triggered.

If Pgp4 does not control the production or localization of CteG, then CteG and Pgp4 may function independently to promote host cells lysis. If this was the case, then a *C. trachomatis* strain lacking both CteG and Pgp4 would be more defective in host cells lysis than strains lacking only CteG or Pgp4. Alternatively, CteG and Pgp4 may act on the same pathway to promote host cell lysis. In this scenario a Pgp4-regulated gene could influence CteG activity, and the double mutant would be indistinguishable from the single mutants in its ability to promote host cell lysis. To analyze this, we used *C. trachomatis* L2/434 or *cteG::aadA*-derived strains carrying Pgp4 but not CteG (in pVector[Pgp4^+^], a recombinant derivative of the endogenous virulence plasmid; **Table 1** and **Supplementary Figures S6, S7**), or neither Pgp4 nor CteG (in pVector[Pgp4^-^], a derivative of pVector[Pgp4^+^] with *pgp4* deleted; **Table 1** and **Supplementary Figures S6, S7**). When analyzing these strains for the ability to generate infectious particles, the ones lacking CteG and/or Pgp4 revealed a defect relative to the strain carrying chromosomally encoded *cteG* and plasmid-encoded *pgp4* (**Supplementary Figure S9A**). This defect was more pronounced for the strain lacking both CteG and Pgp4 (**Supplementary Figure S9A**). Initial experiments also indicated that, for unknown reasons, the levels of host cell lysis mediated by *C. trachomatis* strains (L2/434 and *cteG::aadA*) carrying pVector[Pgp4^+^] were higher than in similar strains carrying the endogenous virulence plasmid (**Supplementary Figure S9B**). As a consequence, the difference between the IFUs released by cells infected by the L2/434 and *cteG::aadA* strains carrying pVector[Pgp4^+^] was less pronounced than in cells infected by similar strains carrying the native plasmid (**Supplementary Figure S9C**). Although this difference in released IFUs is still detectable and can be complemented (**Supplementary Figures S9C, D**), in subsequent experiments analyzing strains carrying pVector[Pgp4^+^] or pVector[Pgp4^+^]-derived plasmids we focused on the more robust monitoring of LDH release into the supernatant of infected cells as a measure of the ability of CteG to mediate chlamydial egress by host cell lysis. HeLa cells were then infected at a MOI of 0.3, for 72 h, by *C. trachomatis* L2/434 carrying pVector[Pgp4^+^] (CteG^+^/Pgp4^+^), L2/434 carrying pVector[Pgp4^-^] (CteG^+^/Pgp4^-^), *cteG::aadA* carrying pVector[Pgp4^+^] (CteG^-^/Pgp4^+^), or *cteG::aadA* carrying pVector[Pgp4^-^] (CteG^-^/Pgp4^-^). This further confirmed that both CteG and Pgp4 contribute to *C. trachomatis*-mediated host cell lysis (**Figure 5A**). However, the CteG^-^/Pgp4^-^ strain showed a defect in host cell lysis similar to the CteG^+^/Pgp4^-^ or CteG^-^/Pgp4^+^ strains (**Figure 5A**). These results indicate that CteG and Pgp4 act on the same pathway to promote host cell lysis mediated by *C. trachomatis*.

### Over-Production of CteG-2HA Suppresses the Defect of CteG- and Pgp4-Deficient *C. trachomatis* to Mediate Host Cell Lysis

CteG can be detected in the cytoplasm of host cells infected by *C. trachomatis* from 16 h post-infection (Pais et al., 2019), but the CteG-dependent increase in host cell lysis occurs from 48 to 72 h post-infection (**Figure 3** and **Supplementary Figure S4**). This suggests a mechanism that initially keeps CteG inhibited in its ability to promote host cell lysis. One hypothesis to explain how CteG and Pgp4 act on the same pathway to promote host cell lysis would be that the product of a Pgp4-regulated gene could be involved in a process of activation or inhibition relief of CteG. We reasoned that if the product of a Pgp4-regulated gene mediates activation or inhibition relief of CteG, then this might be surpassed by overproduction of CteG-2HA. Our previous data indicate that in a *C. trachomatis* strain with plasmid-encoded CteG-2HA its mRNA levels are ~10-fold higher than of chromosomal *cteG* (Pais et al., 2019). Therefore, we infected HeLa cells with the same strains as before (*C. trachomatis* L2/434 carrying pVector[Pgp4^+^] (CteG^+^/Pgp4^+^), L2/434 carrying pVector[Pgp4^-^] (CteG^+^/Pgp4^-^), *cteG::aadA* carrying pVector[Pgp4^+^] (CteG^-^/Pgp4^+^), or *cteG::aadA* carrying pVector[Pgp4^-^] (CteG^-^/Pgp4^-^); **Figure 5A**) but also with Pgp4^+^ and Pgp4^-^ strains carrying a plasmid encoding CteG-2HA (*cteG::aadA* carrying plasmid CteG-2HA[Pgp4^+^] (CteG-2HA^+^/Pgp4^+^), corresponding to the complemented strain used in other experiments, or *cteG::aadA* carrying plasmid CteG-2HA[Pgp4^-^] (CteG-2HA^+^/Pgp4^-^) (**Table 1**), and monitored the release of LDH at 72 h post-infection. This further confirmed that the defect in host cell lysis of the CteG^-^/Pgp4^-^ strain is similar to the CteG^+^/Pgp4^-^ or CteG^-^/Pgp4^+^ strains (**Figure 5B**) and revealed that the Pgp4^-^ strain carrying plasmid-encoded CteG-2HA displays an ability to promote host cell lysis identical to the CteG^+^/Pgp4^+^ and CteG-2HA^+^/Pgp4^+^ strains (**Figure 5B**). Therefore, overproduction of CteG-2HA can compensate for the lack of Pgp4 regarding the ability of *C. trachomatis* to induce host cell lysis.

## Discussion

Understanding how *C. trachomatis* effectors mediate the different stages of the chlamydial infectious cycle is key to understand how this pathogen survives and replicates within host cells. Most studies on chlamydial effectors have focused on those involved in the initial steps of host cell infection and on Incs (Bugalhão and Mota, 2019), including those that mediate exit by extrusion (Lutter et al., 2013; Nguyen et al., 2018; Shaw et al., 2018). In this study, we found that the T3S effector CteG (Pais et al., 2019) is involved in the lytic exit of *C. trachomatis* from host cells. This is the first chlamydial T3S effector involved in this process, thus filling the gap of the previously proposed link between the *C. trachomatis* virulence plasmid and its T3S system (Yang et al., 2015) in mediating this essential step of the chlamydial infectious cycle. Our work, together with previous related studies (Hybiske and Stephens, 2007; Yang et al., 2015), indicates that, as chlamydial egress by extrusion of the inclusion (Lutter et al., 2013; Nguyen et al., 2018; Shaw et al., 2018; Zuck and Hybiske, 2019), *C. trachomatis* lytic exit involves different chlamydial players [CteG, Pgp4, at least one of the several Pgp4-regulated genes (Song et al., 2013; Patton et al., 2018), and CPAF], likely host cell factors and different layers of regulation.

A limitation of our study is that the *cteG::aadA* mutant strain is not isogenic to the parental L2/434 strain and displays a slight growth defect that is CteG-independent. By comparison to the L2/434 strain, and besides the inactivation of *cteG*, we found six nucleotide differences in the CteG-deficient strain leading to four missense mutations and two alterations in non-coding regions. It is presently unclear which of these mutations leads to the slight growth defect of the mutant strain. As detailed in **Supplementary Table S3**, the missense mutations are in a putative lipoprotein (CT734), in a putative integral membrane protein (CT853), and in two known virulence proteins: a T3S chaperone (CT862/LcrH/Scc3) (Fields et al., 2005), and an Inc (CT618) (Bugalhão and Mota, 2019). However, as the defect of the CteG-deficient strain in promoting host cell lysis can be complemented, this confirms the role of CteG in this process.

While the mechanistic details need to be experimentally tested, and other possibilities for how CteG mediates host cell lysis might be envisioned, we propose a hypothetical model in which generation of a CteG protein capable of mediating host cell lysis requires Pgp4-dependent activation, a step that can be suppressed by overproduction of CteG (**Figure 5C**). As *cteG* expression is not regulated by Pgp4 (Carlson et al., 2008; Song et al., 2013; Patton et al., 2018), which we confirmed also at the level of CteG production and localization in infected host cells, the product of at least one Pgp4-regulated gene should be involved in this activation of CteG. At the present, we cannot discriminate whether the hypothetical Pgp4-dependent activation of CteG occurs within chlamydiae or after delivery of CteG into the host cell cytoplasm. In any event, as CteG is type III secreted into the cytoplasm of host cells from at about 16-20 h post-infection and as host cell lysis is only detected from 48 h p.i, there should be an inhibitor in infected host cells that prevents CteG-mediated host cell lysis until later in the chlamydial infectious cycle (**Figure 5C**). This inhibitor could be another chlamydial effector or a host cell factor. CPAF protease is another *C. trachomatis* protein that has been shown to be involved in chlamydial lytic exit (Yang et al., 2015). It is tempting to speculate that CPAF could cleave the hypothetical inhibitor of CteG, thus liberating CteG to exert its lytic action. However, there is conflicting evidence on whether CPAF is secreted into the host cell cytoplasm during the chlamydial infectious cycle or if it is only released after inclusion rupture later in the cycle (Prusty et al., 2018; Bugalhão and Mota, 2019). Furthermore, inhibition of CPAF activity after laser-mediated rupture of the chlamydial inclusion membrane does not prevent subsequent host cell lysis (Kerr et al., 2017). Therefore, how and if CPAF might contribute to *C. trachomatis* lytic exit mediated by CteG remains to be directly analyzed.

Late-stage host cell lysis is a *C. trachomatis*-induced process that initiates with rupture of the inclusion membrane in a chlamydial-dependent manner and culminates with destruction of the host plasma membrane (Hybiske and Stephens, 2007; Yang et al., 2015; Kerr et al., 2017), a mechanism used by other pathogens (Andreadaki et al., 2018; Flieger et al., 2018). Therefore, either directly, or indirectly by activating other chlamydial proteins and/or host cell factors, CteG could promote lysis of the inclusion or host cell plasma membranes. At present, it is not possible to discriminate between the different possibilities. As CteG concentrates at the plasma membrane at late stages of infection, a possible action on the integrity of the plasma membrane to mediate host cell lysis would appear more likely. On the other hand, it has been shown that laser-mediated rupture of the chlamydial inclusion leads to a rapid necrotic cell death-dependent pathway that appears to be mostly mediated by the host (Kerr et al., 2017). The latter has been suggested by the inefficacy of bacterial protein synthesis inhibitors added at 24 h post-infection to prevent host cell lysis after laser-mediated inclusion rupture (Kerr et al., 2017). However, bacterial proteins, such as CteG, already present in the host cell cytoplasm at 24 h post-infection could still mediate plasma membrane rupture if activated or relieved from inhibition upon inclusion rupture. To clarify these aspects, it will be crucial to determine host cells proteins CteG might interact with and to elucidate if it has an enzymatic activity. For example, cysteine proteases have been proposed to mediate chlamydial lytic exit by promoting rupture of the inclusion membrane (Hybiske and Stephens, 2007). While CteG does not seem to possess in its amino acid sequence any consensus motif characteristic of such proteases, it could have a yet undescribed cysteine protease domain. Furthermore, it is also conceivable that CteG could activate a host cell protease, such as calpains that have been suggested to be involved in inclusion membrane rupture (Kerr et al., 2017).

There are reports indicating that *C. trachomatis* LGV strains (such as the strain we used in our study) and *C. muridarum* (a *Chlamydia* species infecting rodents) are prone to be more lytic than ocular and urogenital *C. trachomatis* strains that would exit predominantly by extrusion (Todd and Caldwell, 1985; Yang et al., 2015). In contrast, in another study, the rate of host lysis was reported identical in cells infected by LGV or urogenital strains (Hybiske and Stephens, 2007). While chlamydial lytic exit should enable a rapid re-infection of host cells, exit by extrusion should facilitate dissemination and the release of chlamydiae that remain infectious for longer (Zuck et al., 2017). To understand the advantages of each egress pathway, it would be interesting to correlate possible differences in these processes between *C. trachomatis* serovars or amongst *Chlamydia* species to the genetic and transcriptomic variability we previously described for *cteG* (Pais et al., 2019). Furthermore, at the present, it is unknown if the rate of extrusion is altered in CteG-deficient *C. trachomatis*. Likewise, it is also unknown if the rate of lytic exit is affected in chlamydiae deficient for Incs (CT228 and MrcA) that mediate extrusion (Lutter et al., 2013; Nguyen et al., 2018; Shaw et al., 2018). Future clarification of these aspects could also help understanding the advantages of both exit pathways and how are they controlled by *C. trachomatis*.

Host cell lytic exit is conserved in *Chlamydiae* as this was observed for *Simkania negevensis*, a *Chlamydia*-like microorganism and an emerging pathogen (Koch et al., 2020). However, a simple BLAST analysis indicates there is no CteG homologue in the genome of *S. negevensis*, which suggests that *C. trachomatis* and *S. negevensis* evolved different host cell lytic exit mechanisms. In fact, this is not surprising as the infectious cycle of *S. negevensis* takes much longer than the one of *C. trachomatis*, with infectious forms appearing only about three days after infection (Kahane et al., 2002).

In summary, we describe a new function for the *C. trachomatis* T3S effector CteG in mediating host cell lysis and concomitant chlamydial exit. We propose that CteG achieves this through a cascade of events in which at least a Pgp4-regulated gene participates. This contributes to a better understanding of chlamydial molecular pathogenesis. Despite this, many questions remain to be answered about the general function(s) of CteG and how it promotes host cell lysis. In general, it is unknown how CteG is targeted to the Golgi and plasma membrane in infected host cells, how this is controlled during infection, and if this dual localization corresponds to distinct functions. More specifically, it is unknown if a particular localization of CteG is required for its ability to induce host cell lysis, and so far no chlamydial or host cell proteins interacting with CteG have been identified. Furthermore, as already mentioned, the biochemical activity enabling CteG to mediate host cell lysis is unknown. It also unclear if CteG mediates host cell lysis directly or indirectly. Answering most of these questions is required to provide detailed mechanistic insights on how the action of CteG leads to host cell lysis. Other important questions that remain to be answered include the fine-tuning of CteG activation or availability with the timing of host cell lysis specifically at late stages of infection and the role of Pgp4, Pgp4-regulated proteins and CPAF in these events. Clarification of all these issues may help in ideas to generate genetically modified and attenuated chlamydiae that could be used in vaccine research.

## Author’s Note

While this manuscript was under revision, Bishop and Derré reported that C. trachomatis Inc CTL0390 is also involved in mediating chlamydial host cell lytic exit (Bishop and Derré, 2022).

## Data Availability Statement

The *C. trachomatis* genomic sequences determined in this work are deposited in the European Nucleotide Archive (ENA) under the BioProject accession number PRJEB51643. All the other original contributions presented in the study are included in the article/**Supplementary Material**. Further inquiries can be directed to the corresponding author.

## Author Contributions

IP, SP, and VB performed the experiments and interpreted data. MB and JG contributed to the design of the project and interpreted data. LM conceived and designed the project and interpreted data. IP and LM wrote the manuscript. All authors contributed to manuscript revision, read, and approved the submitted version.

## Funding

This work was supported by Fundação para a Ciência e Tecnologia (FCT) through grant PTDC/BIA-MIC/28503/2017, and in the scope of the projects UIDP/04378/2020 and UIDB/04378/2020 of the Research Unit on Applied Molecular Biosciences – UCIBIO, and LA/P/0140/2020 of the Associate Laboratory Institute for Health and Bioeconomy - i4HB. ISP and SVP were supported by PhD fellowships SFRH/BD/129756/2017 and PD/BD/52210/2013, respectively, also funded by FCT. SVP PhD fellowship was within the scope of the PhD program Molecular Biosciences (PD/00133/2012), funded by FCT.

## Conflict of Interest

The authors declare that the research was conducted in the absence of any commercial or financial relationships that could be construed as a potential conflict of interest.

## Publisher’s Note

All claims expressed in this article are solely those of the authors and do not necessarily represent those of their affiliated organizations, or those of the publisher, the editors and the reviewers. Any product that may be evaluated in this article, or claim that may be made by its manufacturer, is not guaranteed or endorsed by the publisher.
