# Supplementary material for: The Type III Secretion Effector CteG Mediates Host Cell Lytic Exit of Chlamydia trachomatis
Source: Front Cell Infect Microbiol. 2022 Jul 8;12:902210. doi: 10.3389/fcimb.2022.902210 (PMC9318579; doi:10.3389/fcimb.2022.902210)
Supplement: Supplementary file 1 [file DataSheet_1.pdf]

SUPPLEMENTAL MATERIAL

The type III secretion effector CteG mediates host cell lytic exit of *Chlamydia trachomatis*

Inês Serrano Pereira[1,2], Sara Vilela Pais[2†], Vítor Borges[3], Maria José Borrego[3], João Paulo Gomes[3], and Luís Jaime Mota[1,2*]

[1]Associate Laboratory i4HB - Institute for Health and Bioeconomy, NOVA School of Science and Technology, NOVA University Lisbon, 2819-516 Caparica, Portugal.

[2]UCIBIO – Applied Molecular Biosciences Unit, Department of Life Sciences, NOVA School of Science and Technology, NOVA University Lisbon, 2819-516 Caparica, Portugal.

[3]Bioinformatics Unit, Department of Infectious Diseases, National Institute of Health Dr. Ricardo Jorge (INSA), Lisbon, Portugal.

***Corresponding author:**
E-mail: ljmota@fct.unl.pt

[†] **Present Address:** Interfaculty Institute of Microbiology and Infection Medicine (IMIT), Faculty of Medicine, University of Tübingen, Tübingen, Germany

SUPPLEMENTARY TABLE S1. Plasmids used in this study.

Plasmid	Characteristics and construction	Source/Ref.
p2TK2-SW2	*E. coli* - *C. trachomatis* shuttle vector which enables the expression of proteins in *C. trachomatis* (Amp^R).	(Agaisse and Derré, 2013).
pSVP247/pVector[Pgp4$^+$]	Derivative of p2TK2-SW2 for expression of proteins with a C-terminal double hemagglutinin (2HA) tag. Contains the terminator of the *incDEFG* operon (T_{incD}) of *C. trachomatis* L2/434 Bu (Amp^R).	(Da Cunha et al., 2017).
pSVP264/pCteG-2HA/pCteG-2HA[Pgp4$^+$]	Derivative of pSVP247/ pVector[Pgp4$^+$] for the over-expression of CteG-2HA under the control of the predicted *cteG* promoter (P_{cteG};Amp^R).	(Pais et al., 2019).
pIP40/pCteG	Enables the expression of native $CteG_{FL}$ under the control of P_{cteG}. Contains the T_{incD} sequence. A DNA fragment comprising P_{cteG}-*cteG* was amplified from pSVP264/pCteG-2HA using primers 1680 and 2321. Another DNA fragment containing the T_{incD} sequence was amplified from pSVP247/pVector[Pgp4$^+$] with primers 2320 and 1483. Both fragments were then fused by overlapping PCR using primers 1680 and 1483. The resulting DNA product was digested with KpnI and SalI and inserted into those sites of p2TK2-SW2 (Amp^R).	This work.
pIP53/pFabI-CteG-CTL0361	Enables the expression of CTL0359 (FabI), CTL0360 (CteG) and CTL0361. A DNA fragment containing the DNA sequences of *ctl0360* (*cteG*), and of its neighboring genes *ctl0359* and *ctl0361* plus some nucleotides upstream and downstream these genes was amplified from *C. trachomatis* L2/434 chromosomal DNA using primers 2388 and 2389. The resulting DNA product was digested with KpnI and SalI and inserted into those sites of p2TK2-SW2 (Amp^R).	This work.
pIP54/pFabI-CteG	Enables the expression of CTL0359 (FabI) and CTL0360 (CteG). A DNA fragment containing *ctl0359* and *ctl0360* was amplified from *C. trachomatis* L2/434 chromosomal DNA using primers 2388 and 2321. A DNA fragment containing the sequence of T_{incD} was amplified from pSVP247 with primers 2320 and 1483. Both DNA fragments were then fused by overlapping PCR using primers 2388 and 1483. The resulting DNA product was digested with KpnI and SalI and inserted into those sites of p2TK2-SW2 (Amp^R).	This work.
pIP68/ pVector[Pgp4$^-$]	Derivative of pSVP247/pVector[Pgp4$^+$] where *pgp4*/*porf6* is deleted. pSVP247/pVector[Pgp4$^+$] was amplified using primers 2554 and 2555. The resulting DNA product was digested with AscI and ligated, enabling plasmid circularization.	This work.
pIP69/ pCteG-2HA[Pgp4$^-$]	Enables the expression of CteG-2HA under the control of P_{cteG} in a *pgp4*$^-$ background. A DNA fragment containing P_{cteG}-*cteG* was amplified from pSVP264/pCteG-2HA using primers 1680 and 1552. The resulting DNA product was digested with KpnI and NotI and inserted into those sites of pIP68/pVector[Pgp4$^-$] (Amp^R).	This work.

[a]Amp^R: ampicillin resistance; Kan^R: kanamycin resistance

SUPPLEMENTARY TABLE S2. DNA primers used in this study.

Number	Description	Sequence (5’ → 3’)
383	CT105_seq; forward primer to verify the presence of the transformation plasmid encoding CteG-2HA in *C. trachomatis* strains.	AAGCTCCAAGAGTTATTGG
1479	p2TK2-SW2_seq_Rv; reverse primer to verify the presence of the transformation plasmid encoding CteG-2HA in *C. trachomatis* strains.	ACAAAATCAAAACAGAATCG
1483	IncDTerm_Rv_SalI; reverse primer to construct pIP40/pCteG and pIP54/pFabI-CteG.	GATCGTCGACGTCTTAGGAGCTTTTTGCAATGC
1552	CT105_NotI_Rv; forward primer to construct pIP69/ pCteG-2HA[Pgp4^{-}].	GATCGCGGCCGCGGATAGAGGAGCTTTGCACACC
1680	P_CT105_KpnI_p2TK2_Fwd; forward primer to construct pIP40/pCteG and pIP69/ pCteG-2HA[Pgp4^{-}].	GATCGGTACCTTCTTTATTATTGAGAAACG
1865	GII_R_test; reverse primer to verify the presence of a group II intron within *cteG* in *C. trachomatis* strains.	TCTCGGAGTATACGGCTCTG
1934	CT105-C; forward primer used to verify the presence of a group II intron within *cteG* in *C. trachomatis* strains.	ATGGAGCCGTTTGTGTGGTT
2173	pSUmC Seq_4; forward primer to verify the deletion of *pgp4* in *C. trachomatis* strains.	TGCGGCCCTAGAATTTGG
2320	CT105_IncDTerm_OL_F; forward overlap primer to construct pIP40/pCteG and pIP54/pFabI-CteG.	GGTGTGCAAAGCTCCTCTATCTAGGGATGACATGTGATTCGCG
2321	CT105_IncDTerm_OL_R; reverse overlap primer to construct pIP40/pCteG and pIP54/pFabI-CteG.	CGCGAATCACATGTCATCCCTAGATAGAGGAGCTTTGCACACC
2388	FabI_Fw_KpnI; forward primer to construct pIP53/pFabI-CteG-CTL0361 and pIP54/pFabI-CteG.	GATCGGTACCAAAATTGTTATACAGACGGC
2389	CTL0359_Rv_SalI; reverse primer to construct pIP53/pFabI-CteG-CTL0361.	GATCGATGTCGACCGAGAATAATAACCCAGCCC
2554	Pgp4_del_Rv_AscI; reverse primer to construct pIP68/ pVector[Pgp4^{-}].	GATCGGCGCGCCAAGGCTGAATAGACAACTTACTC
2555	Pgp4_del_Fw_AscI; forward primer to construct pIP68/pVector[Pgp4^{-}].	GATCGGCGCGCCAATTTTGCATAACAAACCCCGTAATTC
2631	Seq_pgp4_Rv; reverse primer to verify the deletion of *pgp4* in *C. trachomatis* strains.	GTGGTATGGGTTAATGCCC

SUPPLEMENTARY TABLE S3. Nucleotide changes between *C. trachomatis* strains L2 434/Bu (*cteG*$^+$) and *cteG::aadA* (*cteG*$^-$) used in this study.

Reference genome position[1]	Base pair change	Amino acid change	Variant type[2]	Nt in reference strain[3]	L2/434/Bu locus tag[4]	Serovar D locus tag[5]	Gene	Gene product	L2/434/Bu (*cteG*$^+$) (frequency)	*cteG::aadA* (*cteG*$^-$) (frequency)
127,340	*C659T*	A220V	Missense	C	CTL0103	CT734		Putative lipoprotein	C (100%)	T (100%)
282,299	*C11T*	S4L	Missense	G	CTL0226	CT853		Putative integral membrane protein	G (100%)	A (100%)
295,554	*G592T*	A198S	Missense	C	CTL0237	CT862	*lcrH*	Type III secretion chaperone	C (90%)<br>A (10%)	A (100%)
929,598	C→A		Noncoding	C	Upstream CTL0805 /CTL0806		*hisS /uhpC*	*hisS*: histidyl-tRNA synthetase; *uhpC*: putative sugar phosphate permease	C (84%)<br>A (16%)	A (100%)
929,717	A→C		Noncoding	C	Upstream CTL0805 /CTL0806	Upstream CT543 /CT544	*hisS /uhpC*	*hisS*: histidyl-tRNA synthetase; *uhpC*: putative sugar phosphate permease	A (66%); C(34%)	C (100%)
1,017,120	*C391A*	R131S	Missense	C	CTL0882	CT618		Putative membrane protein	C (58%)<br>A (25%)<br>T (17%)	A (100%)

[1] Nucleotide position in *C. trachomatis* L2/434/Bu reference strain genome (GenBank accession number AM884176.1).
[2] Single nucleotide variant classification: Noncoding – outside coding sequence; Missense – substitution.
[3] Nucleotide described for *C. trachomatis* L2/434/Bu reference strain.
[4] Locus designation in *C. trachomatis* L2/434/Bu reference strain.
[5] Locus designation in *C. trachomatis* D/UW-3/CX (Serotype D) reference strain (GenBank accession number AAC68146.1).

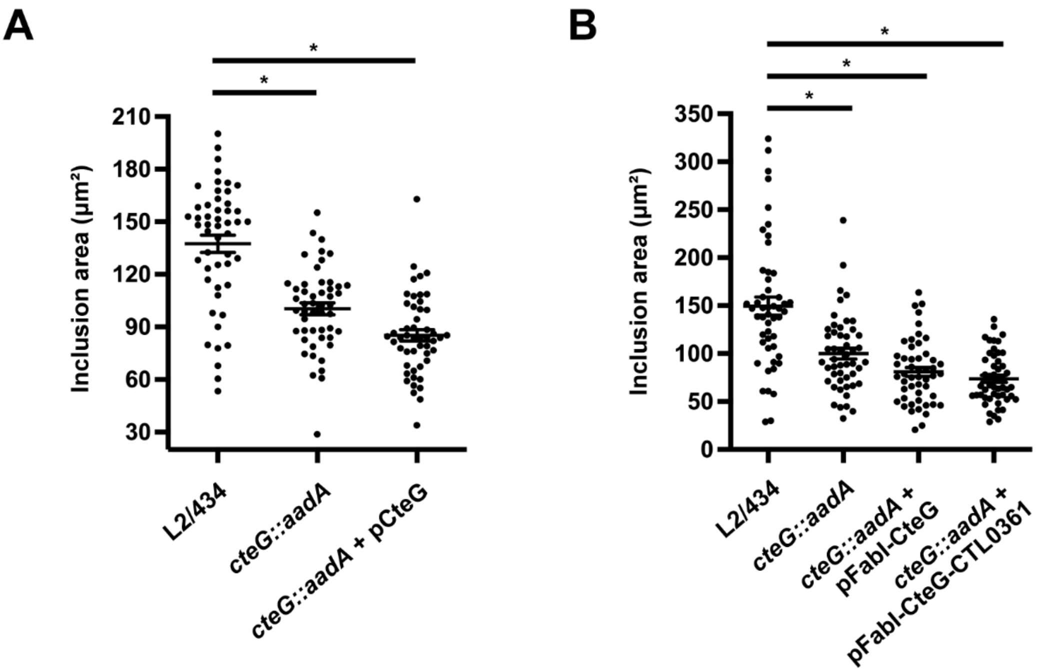

Supplementary Figure S1 – Plasmids encoding native *cteG* or *cteG* and its flanking genes do not complement the growth defect of the *Chlamydia trachomatis cteG::aadA* strain. HeLa cells were infected for 24 h with the indicated *C. trachomatis* strains. Cells were then fixed with methanol and immunolabelled with an antibody against *C. trachomatis* major outer membrane protein (MOMP) and with the appropriate fluorophore-conjugated secondary antibody. Independent photos of infected cells were obtained by immunofluorescence microscopy and the area of 50 randomly chosen inclusions was measured using the software Fiji. **(A)** pCteG carries *cteG* alone. (**B)** pFabI-CteG carries *cteG* and *ctl0359/fabI*; pFabI-CteG-CTL0361 carries *cteG*, *ctl0359/fabI* and *ctl0361* (Figure 1A). Statistical significance was determined by using ordinary one-way ANOVA and Dunnett post-test analysis (*p<0.0001).

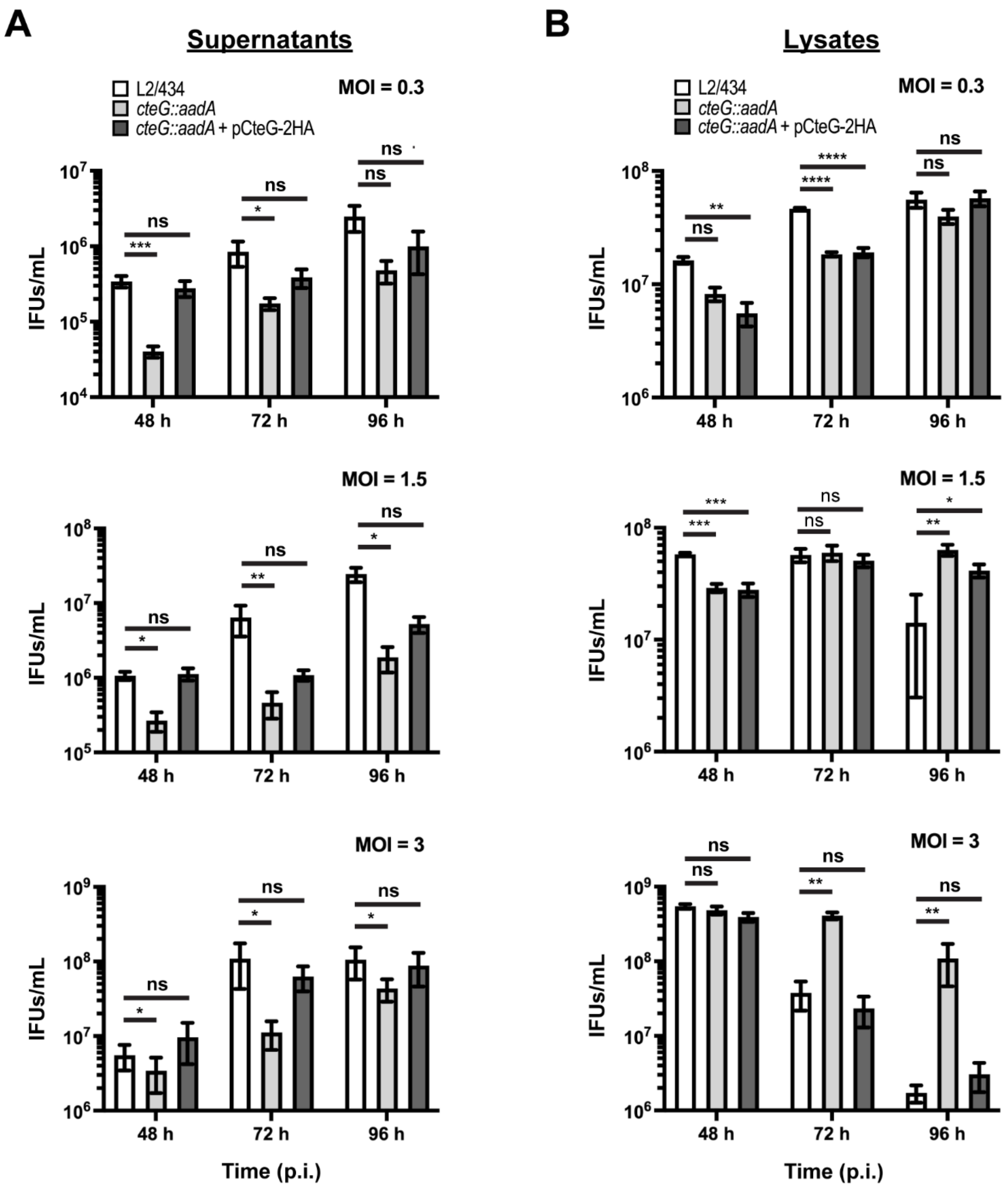

Supplementary Figure S2 – *Chlamydia trachomatis cteG::aadA* displays a CteG-dependent defect in egress from infected host cells. HeLa cells were infected with *C. trachomatis* parental (L2/434), mutant (*cteG::*aadA) and complemented (*cteG::aadA* harboring a plasmid encoding CteG-2HA; pCteG-2HA, also named pCteG-2HA/[Pgp4^{+}]) for 48, 72 or 96 h at the multiplicity of infection (MOI) of 0.3, 1.5 or 3. At each time post-infection (p.i.), supernatant and lysate fractions were collected as described in the Materials and Methods. Fresh layers of HeLa cells were infected with serial dilutions of the supernatant **(A)** or lysate **(B)** fractions, and the number of recoverable inclusion forming units (IFUs) was determined by immunofluorescence microscopy. Data correspond to mean ± standard error of the mean (n≥3). For each time point, statistical significance was determined by using ordinary one-way ANOVA and Dunnett post-test analysis relative to the L2/434 parental strain. For statistical analysis, natural logarithm was applied to data to ensure normality of the populations. (ns, non-significant; *p<0.5; **p<0.01, ***p<0.001; p<0.0001).

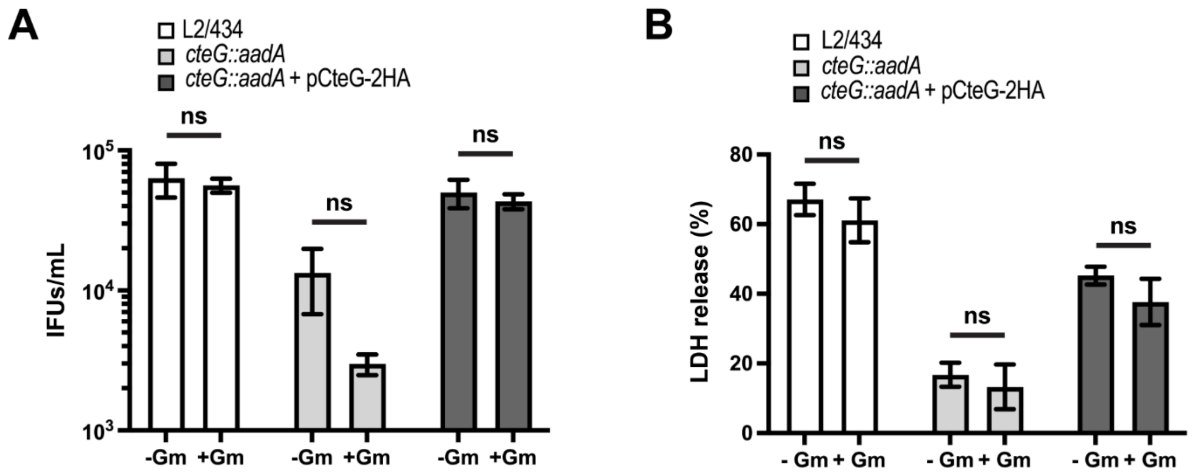

Supplementary Figure S3 – The presence of gentamicin in the infection medium during the first 24 h of infection does not significantly alter the number of released inclusion forming units or the ability of the strains to cause host cell lysis. HeLa cells were infected with *Chlamydia trachomatis* strains L2/434, *cteG::aadA* and *cteG::aadA* harboring a plasmid encoding CteG-2HA (pCteG-2HA, also named pCteG-2HA/[Pgp4^{+}]) with a multiplicity of infection (MOI) of 0.06 for 48 h (A), or a MOI of 0.3 for 72 h (B) and incubated under the presence of 10 µg/mL gentamicin during the first 24 h of infection. At this time point, the medium was replaced by one without gentamicin. **(A)** The supernatant fraction of infected cells was collected and IFUs were quantified by immunofluorescence microscopy. **(B)** The release of host lactate dehydrogenase (LDH) into the supernatant of infected cells was measured using a CytoScan^{TM} LDH Cytotoxicity Assay kit (G-Biosciences). Data in (A) and (B) correspond to the mean $\pm$ standard error of the mean (n=3). Statistical significance was determined for each strain between presence and absence of gentamicin conditions using two-tailed unpaired Student's t-test (ns, non-significant). For statistical analysis in (A), natural logarithm was applied to data to ensure normality of the populations.

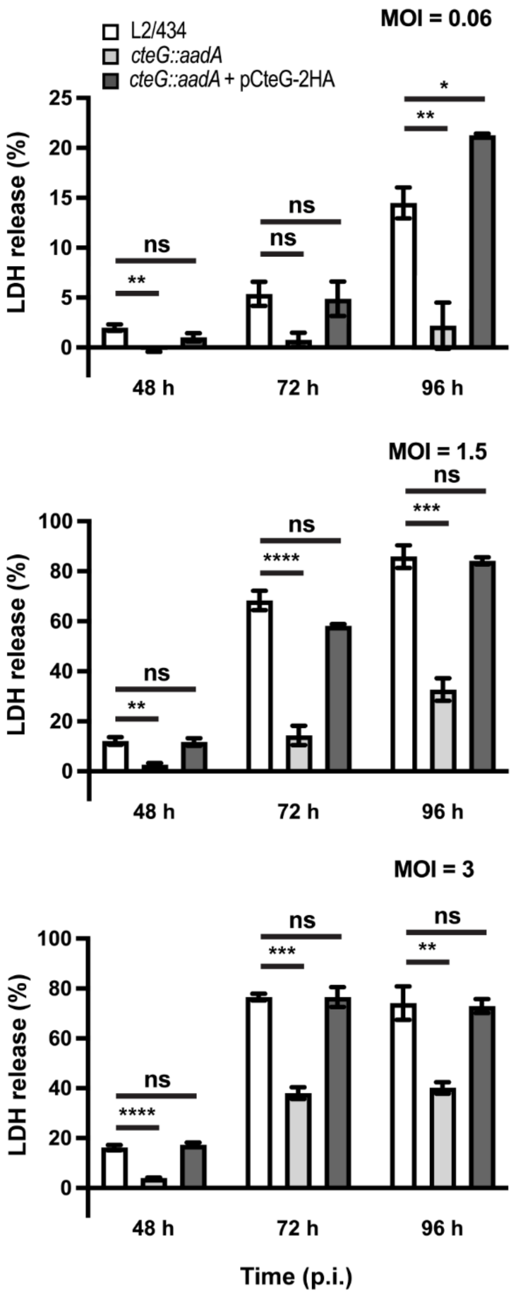

Supplementary Figure S4 – The *Chlamydia trachomatis cteG* mutant strain displays a CteG-dependent host cell lysis defect. HeLa cells were infected with *C. trachomatis* strains L2/434, *cteG::aadA* and *cteG::aadA* harboring a plasmid encoding CteG-2HA (pCteG-2HA, also named pCteG-2HA/[Pgp4^{+}]), using a multiplicity of infection (MOI) of 0.06, 1.5 or 3, for 48, 72 or 96 h. The release of host LDH into the supernatant of infected cells was quantified using a CytoScan^{TM} LDH Cytotoxicity Assay kit (G-Biosciences). Data are representative of five independent experiments and correspond to the mean ± standard error of the mean of three biological replicates. For each time post-infection (p.i.), statistical significance was determined by using ordinary one-way ANOVA and Dunnett post-test analysis relative to the L2/434 parental strain (ns, non-significant; *p<0.05; **p<0.01; ***p<0.001; ****p<0.0001).

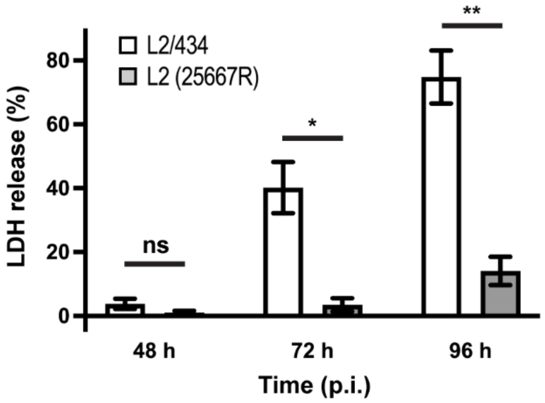

Supplementary Figure S5 – The *C. trachomatis* virulence plasmid contributes to lytic exit from host cells. HeLa cells were infected with *C. trachomatis* L2/434 and with plasmid-deficient 25667R strain for 48, 72 or 96 h at a MOI of 0.3. At each time post-infection (p.i.), the release of host lactate dehydrogenase (LDH) into the supernatant of infected HeLa cells was measured using a CytoScan^{TM} LDH Cytotoxicity Assay kit (G-Biosciences). Data correspond to the mean $\pm$ standard error of the mean of three independent experiments. Statistical significance was determined for each time point by using a two-tailed unpaired Student's t-test (ns, non-significant; *p<0.05; **p<0.01).

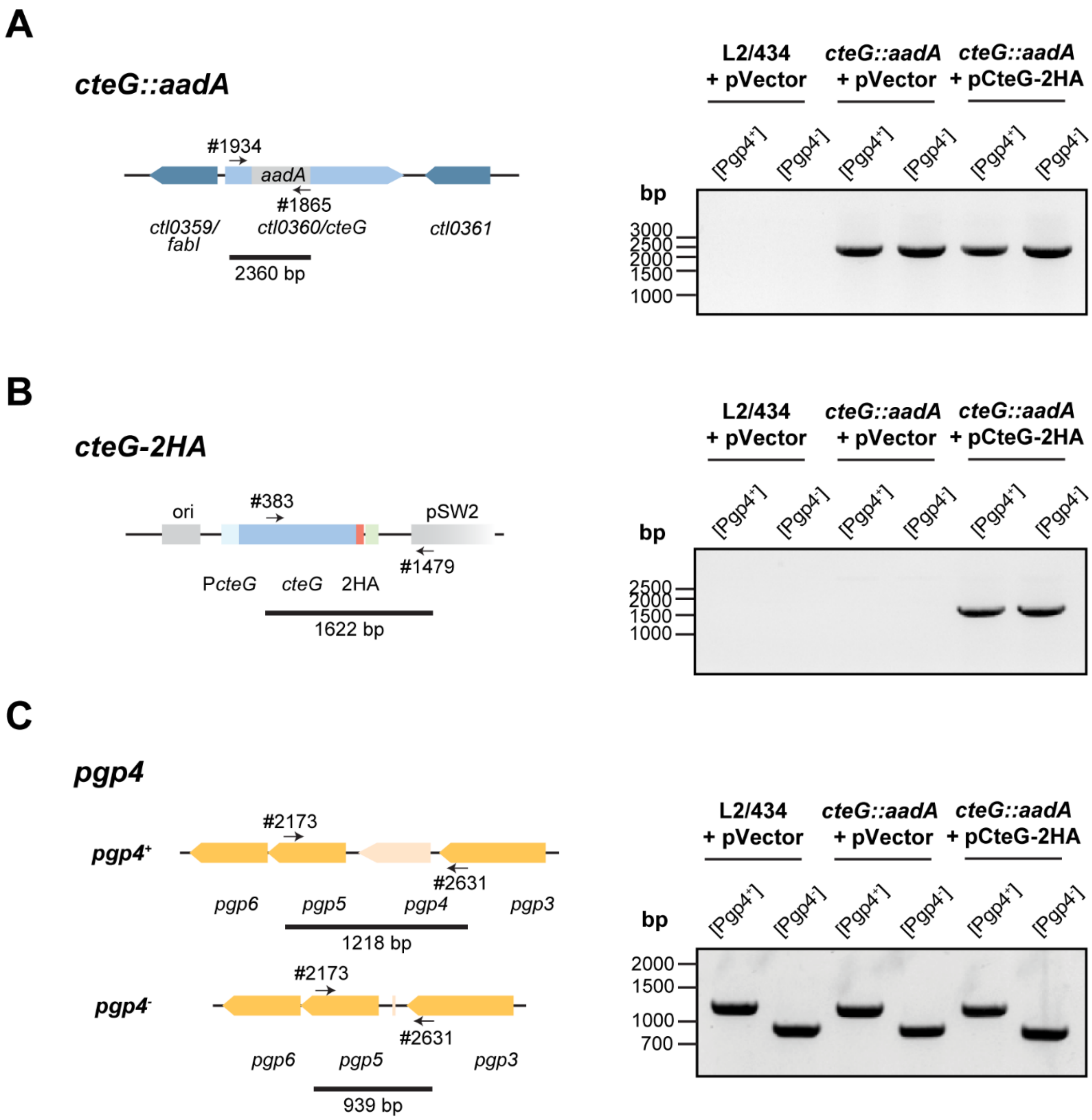

Supplementary Figure S6 – Verification of the accuracy of *Chlamydia trachomatis* strains generated in this work. The indicated *C. trachomatis* strains (see plasmid and strain nomenclature in Table 1) were verified by PCR **(A)** for the presence or absence of a group II intron interrupting *cteG* (*cteG::aadA*), **(B)** for the presence of the plasmid carrying *cteG-2HA*, or **(C)** for the presence or absence of intact plasmid-encoded *pgp4* (*pgp4*). Plasmid pSVP264 is the complementing plasmid used throughout this work (also named pCteG-2HA or pCteG-2HA[Pgp4$^+$]; Table 1 and Supplementary Table S1). Illustrations of all features are depicted. The arrows and numbers indicate the approximate hybridization site of DNA oligonucleotides (Supplementary Table S2) used in PCR reactions, yielding amplification products of the indicated lengths in base pairs (bp).

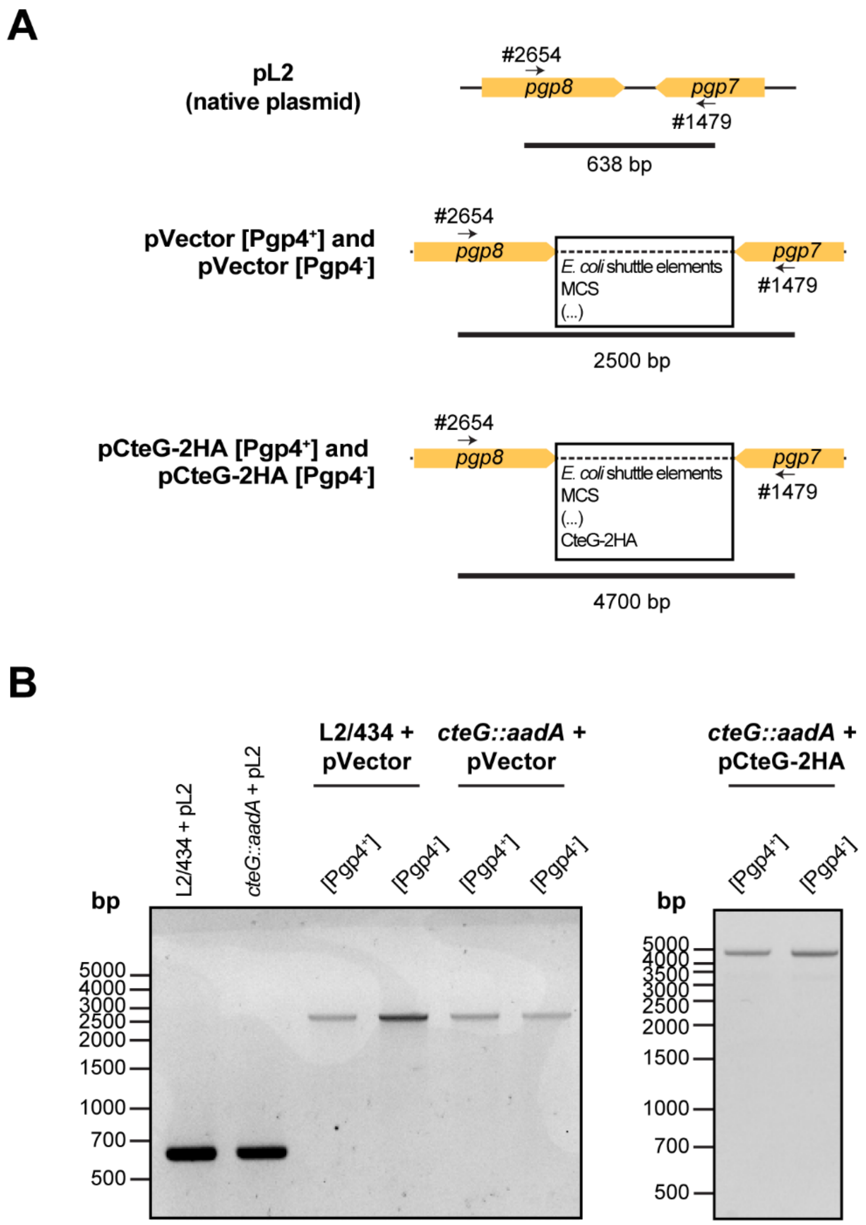

Supplementary Figure S7 – Verification of the replacement of the *Chlamydia trachomatis* native plasmid by recombinant plasmids with or without *pgp4*. (A) DNA organization of the indicated plasmids. The arrows and numbers indicate the approximate hybridization site of DNA oligonucleotides (Supplementary Table S2) used in PCR reactions, yielding amplification products of the indicated lengths in base pairs (bp). **(B)** Presence of the *C. trachomatis* native plasmid (pL2) in L2/434 and *cteG::aadA* strains and its loss and replacement in strains carrying instead plasmids pVector[Pgp4$^+$], pVector[Pgp4$^-$], pCteG-2HA[Pgp4$^+$], or pCteG-2HA[Pgp4$^-$] (Supplementary Table S1) was confirmed by PCR.

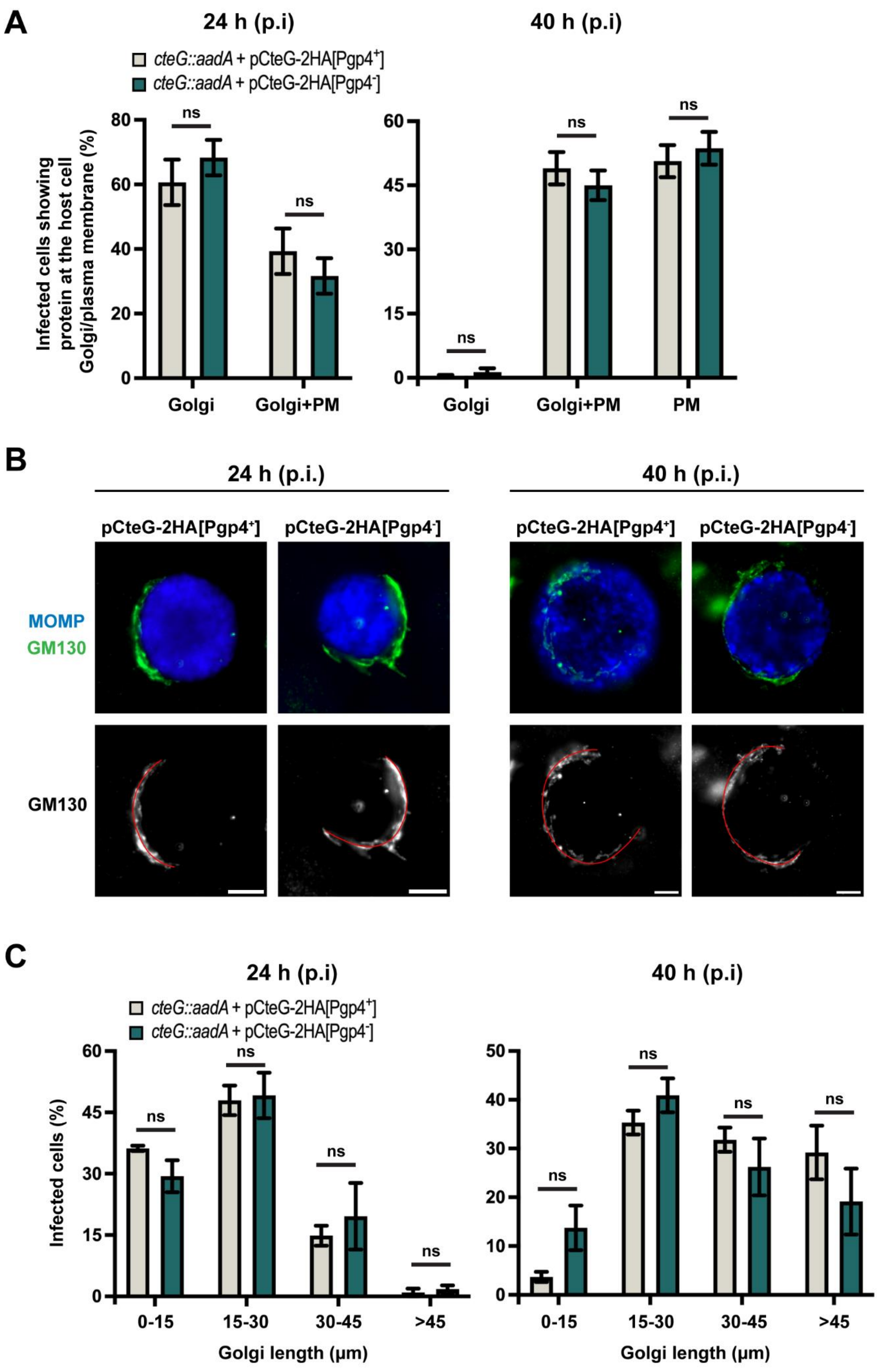
A
24 h (p.i)
40 h (p.i)
cteG::aadA + pCteG-2HA[Pgp4+]
cteG::aadA + pCteG-2HA[Pgp4-]
Infected cells showing protein at the host cell Golgi/plasma membrane (%)
ns
Golgi
Golgi+PM
PM
B
24 h (p.i.)
40 h (p.i.)
pCteG-2HA[Pgp4+]
pCteG-2HA[Pgp4-]
MOMP
GM130
GM130
C
24 h (p.i)
40 h (p.i)
cteG::aadA + pCteG-2HA[Pgp4+]
cteG::aadA + pCteG-2HA[Pgp4-]
Infected cells (%)
0-15
15-30
30-45
>45
Golgi length (µm)

Supplementary Figure S8 – Localization of CteG and Golgi distribution around the inclusion are indistinguishable in cells infected by *Chlamydia trachomatis* Pgp4$^+$ or Pgp4$^-$ strains. HeLa cells were infected by *C. trachomatis* strains harboring pCteG-2HA[Pgp4$^+$] or pCteG-2HA[Pgp4$^-$] at a MOI of 0.3 for 24 or 40 h, fixed with methanol and immunolabelled with antibodies against *cis*-Golgi marker GM130 (green) and HA (red), or anti-MOMP (blue) when used, and with the appropriate fluorophore-conjugated secondary antibodies. **(A)** Fluorescence microscopy was used to enumerate cells showing CteG-2HA only at the Golgi, only at the plasma membrane (PM) or both at the Golgi and at the plasma membrane (Golgi+PM). Data correspond to the mean ± standard error of the mean of three independent experiments (N=100). **(B)** Images of random infected cells were collected by fluorescence microscopy and the extension of Golgi distribution around the inclusion was measured as previously described (Pais et al., 2019), which is exemplified by the red line in the lower panel. Scale bars, 5 µm. **(C)** Percentage of infected cells with Golgi around the inclusion of the indicated length. Data are mean ± standard error of the mean of three independent experiments (N≥35 per condition). In (A) and (B), statistical significance was determined for each time point by using a two-tailed unpaired Student's t-test (ns, non-significant).

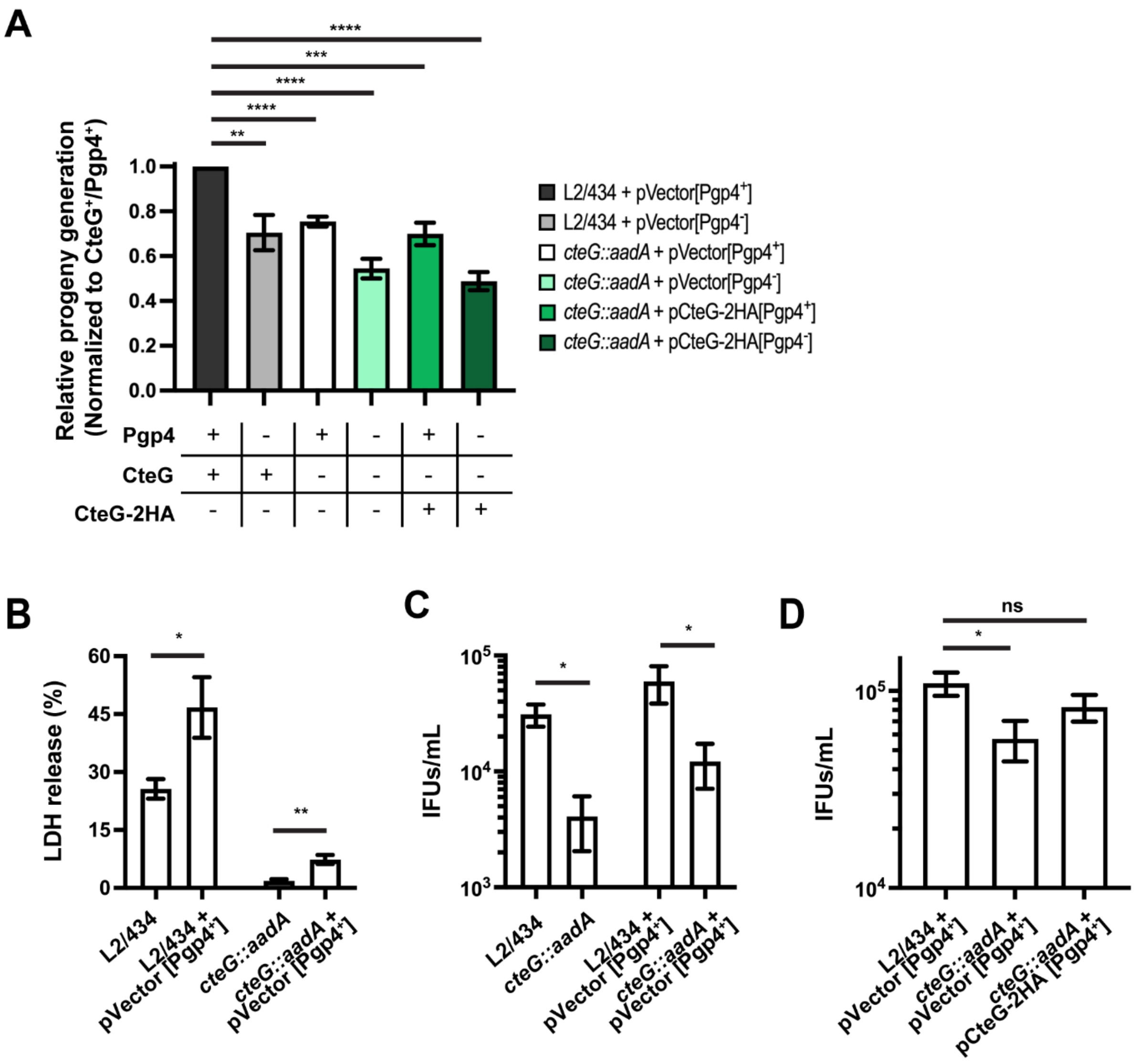

Supplementary Figure S9 – Characterization of *Chlamydia trachomatis* strains used and generated in this study. (A) The ability of the *C. trachomatis* strains used and constructed in this study (see Table 1) to generate infectious progeny was tested, as described in Materials and Methods. In each assay, the values were normalized to that of the $CteG^+/Pgp4^+$ strain. Data correspond to the mean ± standard error of the mean (n=4). **(B, C, and D)** HeLa cells were infected with the indicated *C. trachomatis* with a multiplicity of infection (MOI) of 0.3 for 72 h (B), or a MOI of 0.06 for 48 h (C and D). **(B)** The release of host lactate dehydrogenase (LDH) into the supernatant of infected cells was quantified using a CytoScan™ LDH Cytotoxicity Assay kit (G-Biosciences). Data correspond to mean ± standard error of the mean (n=3). **(C and D)** The number of inclusion forming units (IFUs) in the supernatant fraction was quantified as described in Materials and Methods. Data correspond to mean ± standard error of the mean of three (C) or seven (D) independent experiments. Statistical significance was determined using two-tailed unpaired Student's t-test (A, B, and C) or by using ordinary one-way ANOVA and Dunnett post-test analysis relative to the $CteG^+/Pgp4^+$ strain. In (C) and (D), data was transformed by applying the natural logarithm to ensure normality of the populations. (ns, non-significant; *p<0.05; **p<0.01; ***p<0.001; ****p<0.0001).

SUPPLEMENTAL REFERENCES

Agaisse, H., and Derré, I. (2013). A *C. trachomatis* Cloning Vector and the Generation of *C. trachomatis* Strains Expressing Fluorescent Proteins under the Control of a *C. trachomatis* Promoter. *PLoS ONE* 8. doi: 10.1371/journal.pone.0057090.

Da Cunha, M., Pais, S. V., Bugalhão, J. N., and Mota, L. J. (2017). The *Chlamydia trachomatis* type III secretion substrates CT142, CT143, and CT144 are secreted into the lumen of the inclusion. *PLoS ONE* 12. doi: 10.1371/journal.pone.0178856.

Pais, S. v, Key, C. E., Borges, V., Pereira, I. S., Gomes, J. P., Fisher, D. J., et al. (2019). CteG is a *Chlamydia trachomatis* effector protein that associates with the Golgi complex of infected host cells. *Scientific Reports* 9, 6133. doi: 10.1038/s41598-019-42647-3.
